# Identification of MscS as a Key L‐Glutamate Exporter in *Bacillus methanolicus*

**DOI:** 10.1111/1751-7915.70252

**Published:** 2025-10-22

**Authors:** Luciana Fernandes Brito, Davide Luciano, Marta Irla, David Virant, Gaston Courtade, Trygve Brautaset

**Affiliations:** ^1^ Department of Biotechnology and Food Science Norwegian University of Science and Technology Trondheim Norway; ^2^ Department of Biological and Chemical Engineering Aarhus University Aarhus Denmark; ^3^ Acies Bio d.o.o. Ljubljana Slovenia

**Keywords:** *Bacillus methanolicus*, homoheptamer, l‐glutamate, mechanosensitive channel, methylotroph, RNA sequencing

## Abstract

Small‐conductance mechanosensitive channels (MscS) are established l‐glutamate exporters in industrially relevant bacteria, yet their role in the methylotrophic bacterium *Bacillus methanolicus* , a promising platform for sustainable methanol‐based l‐glutamate production, remains unexplored. Our research on *B. methanolicus* MGA3 identifies its MscS as the sole mechanosensitive channel in this organism and a key exporter of the amino acid l‐glutamate, providing valuable insights into its potential for industrial applications. Transcriptomic analysis of *B. methanolicus* wild type cultured on an l‐glutamate production medium revealed downregulation of the fatty acid biosynthesis genes *fadR*, *fadF*, *mutB2*, and *acdA*, suggesting that fatty acid metabolism is influenced by l‐glutamate overproduction, with consequent changes in membrane fluidity likely driving mechanosensitive channel‐mediated l‐glutamate efflux. The MscS‐like channel in *B. methanolicus* shares structural and functional similarities with MscS in *Escherichia coli* and with MscCG in *Corynebacterium glutamicum* . *In silico* structural predictions show that MGA3 MscS forms a homoheptameric structure with a transmembrane TM‐barrel, resembling that of *E. coli* MscS. The opening mechanism of the channel, driven by membrane dynamics, involves coordinated rotation and flipping of its transmembrane helices, with variations in lipid composition potentially influencing the channel's activity. Additionally, under biotin‐replete conditions, where this essential coenzyme supports carboxylases involved in fatty acid biosynthesis, l‐glutamate overproduction was suppressed in MGA3. Finally, metabolic engineering experiments inducing MscS gain‐ and loss‐of‐function further confirmed the channel's critical role in *B. methanolicus* amino acid production, proportionally enhancing and reducing l‐glutamate efflux, respectively. These findings open doors to novel strategies for engineering *B. methanolicus* and related methylotrophic organisms for sustainable amino acid production.

## Graphical abstract

l‐Glutamate export in the methylotrophic bacterium *Bacillus methanolicus* , driven by an MscS‐like mechanosensitive channel. Figure created with BioRender (https://BioRender.com/t99s28).

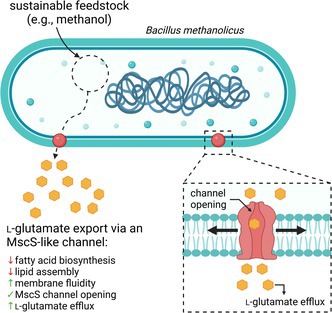

## 1 Introduction

The ability to adapt to environmental changes that disrupt normal cell turgor, such as hypoosmotic shock, is crucial for bacteria. The mechanosensitive channel of small conductance (MscS) gates and releases solutes upon interactions between the channel and lipids in its grooves. Numerous studies, using membrane metrics, show that mechanosensitive channels undergo conformational changes, which are significantly influenced by lipid composition and their specific locations within the membrane (Nakayama, Komazawa, et al. 2018; Cox et al. 2018; Booth et al. 2007). A recent study shows that MscS can be delipidated by detergents and closed by reintroducing lipids, suggesting that lipid extrusion triggers gating (Flegler et al. 2021). These channels have significant potential as exporters of valuable industrial products, leading to extensive characterisation research of the mechanosensitive channels from industrial biotechnology workhorses *Corynebacterium glutamicum* and *Escherichia coli* (Nakayama, Komazawa, et al. 2018; Becker et al. 2012; van den Berg et al. 2016; Malcolm et al. 2015; Kawasaki and Martinac 2020). Moreover, MscS‐like channels share a core architecture, but their gating mechanisms and conductive properties are fine‐tuned based on size and structural differences (Flegler et al. 2020). Therefore, it is worth exploring similar exporter channels in other organisms that show promise for industrial applications.

Methylotrophic bacterial hosts can utilise one‐carbon (C1) compounds such as methanol as their sole source of carbon and energy (Zhang et al. 2018; Schrader et al. 2009; Cotton et al. 2020). Methanol is an attractive second‐generation feedstock due to its abundance, low cost, renewability, and non‐competitiveness with food and feed industries (Zhang et al. 2018; Whitaker et al. 2015), and native methylotrophs have therefore garnered increasing interest for producing a variety of value‐added chemicals (Hakvåg et al. 2020; Sonntag et al. 2015; Brautaset et al. 2007; Irla et al. 2017; Nærdal et al. 2015). Among them, *Bacillus methanolicus* —a Gram‐positive, spore‐forming methylotroph—has emerged as a promising platform organism. The extensively studied MGA3 strain naturally produces high levels of the tricarboxylic acid (TCA)‐cycle‐derived amino acid L‐glutamate in methanol‐based fed‐batch fermentations, reaching titers of up to 60 g L^−1^ (Irla et al. 2017), and has been engineered to produce l‐glutamate derivatives such as γ‐aminobutyrate (Irla et al. 2017). Notably, *B. methanolicus* can also grow on the sugar alcohol mannitol (López et al. 2019; Delépine et al. 2020), a key component of seaweed biomass (Pérez‐García et al. 2022; Hal and Huijgen 2014), enabling cadaverine production from mannitol‐rich brown seaweed extracts (Hakvåg et al. 2020). Together, these traits highlight the organism's potential for flexible, sustainable bioprocesses using both methanol and seaweed‐derived carbon sources.

While MscS channels have been extensively studied in Gram‐negative bacteria, their roles in l‐glutamate export in Gram‐positive species—particularly methylotrophs like *B. methanolicus* —are comparatively less characterised. Moreover, due to the above‐mentioned reasons, *B. methanolicus* is considered a promising host in the biotechnology industry. Understanding the metabolic processes behind its sustainable production of value‐added compounds is essential. For example, the mechanism of l‐glutamate export has not been elucidated yet in this bacterium. l‐Glutamate is a moderately effective compatible solute, and its intracellular concentration rises in *B. methanolicus* in tandem with increasing external osmolarity (Frank et al. 2021). Interestingly, a substantial portion of the newly synthesised l‐glutamate is excreted from the cell (Frank et al. 2021). l‐Glutamate export is further enhanced by supplementing the *B. methanolicus* fermentation medium with surfactants (Schendel et al. 2000). Here, we analysed *B. methanolicus* strain MGA3 using *in silico*, transcriptomics, and physiological studies to investigate the molecular mechanisms underlying l‐glutamate export, a critical step toward optimising *B. methanolicus* biotechnological potential. Our findings identify the MscS as a key l‐glutamate exporter and provide insights into its mode of action.

## 2 Results and Discussion

### 2.1 Media‐Induced Methanol‐Based l‐Glutamate Production in *B. methanolicus* MGA3

This study aims to explore the mechanisms underlying the export of l‐glutamate in *B. methanolicus* . To achieve this, we evaluated the growth of *B. methanolicus* under two different media conditions. The first medium (MVcM) is a commonly employed minimal medium for *B. methanolicus* MGA3 growth (Brito et al. 2021), while the other medium was specifically optimised for l‐glutamate production (MVcMii) (Irla et al. 2025). Under MVcM conditions, the *B. methanolicus* wild‐type strain MGA3 produced 38 ± 2 mg/L l‐glutamate in shake flask, corresponding to 67 ± 3 mg/g CDW. In contrast, growth in MVcMii medium yielded 333 ± 67 mg/g CDW l‐glutamate. The l‐glutamate production titre increased to 526 ± 100 mg/L in the MVcMii medium, representing an approximately 14‐fold enhancement attributable to the altered media composition (Table 1). Despite achieving a similar growth rate in both media, the biomass of MGA3 cultivated in MVcM was approximately 2.8‐fold lower compared to MVcMii (Table 1). This difference can be attributed to the additional carbon source (5 g/L mannitol) present in the MVcMii composition (Table 5).

**TABLE 1:** Growth and l‐glutamate production in *B. methanolicus* MGA3 cultivated in MVcM in comparison to MVcMii medium. *B. methanolicus* cells were cultivated in shake flasks, supernatants were taken at 27 h of cultivation, and their l‐glutamate content was quantified by means of HPLC. Data are represented as means and standard deviations of technical triplicates.

Media	Growth rate (1/h)	Biomass (*g CDW/L)	l‐Glutamate (mg/L)	l‐Glutamate (mg/g CDW)
MVcM	0.30 ± 0.02	0.56 ± 0.01	37 ± 2	67 ± 3
MVcMii	0.26 ± 0.01	1.58 ± 0.03	526 ± 100	333 ± 67

*Note:* * g CDW: grams of cell dry weight.

Methanol‐based l‐glutamate production in MVcM varies between *B. methanolicus* studies. The l‐glutamate titers observed in our flask fermentation under MVcM medium are lower than those reported in similar experiments conducted by Krog et al. (800 mg/L) (Krog et al. 2013). However, our findings surpass those of Frank et al., who reported 19 mg/g CDW, with our study revealing approximately 67 mg/g CDW (Frank et al. 2021). Slight differences in growth conditions may have contributed to this variation. While we routinely used a 10% working volume (liquid‐to‐flask ratio) during cultivation, Krog et al. used 20%, whereas Frank et al. employed a water bath for controlling the temperature of shaking flasks. However, the underlying reasons for this variation require further investigation. For MVcMii, in addition to supplementing the medium with mannitol as an extra carbon source and doubling the concentration of the nitrogen source ((NH_4_)_2_SO_4_), the concentration of the magnesium source (MgSO_4_) is reduced by 80% compared to MVcM. Indeed, l‐glutamate production in *B. methanolicus* was reported to be enhanced by magnesium limitation in the growth medium (Zaręba and Ziarno 2024). Magnesium limitation is a classic trigger for l‐glutamate overproduction in *C. glutamicum* . In the presence of Mg^2+^, glutamate dehydrogenase efficiently assimilates ammonium using NADPH, but under magnesium‐limiting conditions, reduced enzyme activity limits ammonium assimilation and diverts α‐ketoglutarate toward l‐glutamate synthesis (Fahien et al. 1990). This highlights the positive impact of modulating external physiological factors on l‐glutamate production. Notably, *B. methanolicus* MGA3 demonstrates up to a 10‐fold increase in l‐glutamate efflux under osmotic changes (Frank et al. 2021). In bacteria, the presence of the non‐ionic emulsifier Tween 80 in the growth medium significantly impacts the fatty acid profile; it can increase saturated fatty acids and reduce unsaturated fatty acids, leading to changes in the composition and function of bacterial cell membranes (Zaręba and Ziarno 2024; Mohammad and Taha Daod 2021). The addition of Tween 80 in the fed‐batch fermenter is known to trigger the production of 57 g/L l‐glutamate in this organism in comparison to the 24 g/L l‐glutamate produced when no surfactant was added (Schendel et al. 2000). In response to environmental fluctuations, bacterial cell walls may feature the activation of mechanosensitive channels by lowered membrane tension. These channels serve not only as osmotic safety valves to prevent cell bursting during hypoosmotic shocks. Through a pump‐and‐leak mechanism, they continuously release l‐glutamate, contributing to the regulation of cellular turgor pressure (Nakayama, Hashimoto, et al. 2018; Levina et al. 1999).

### 2.2 Pyrimidine Biosynthesis, Sugar Alcohol Catabolism and Flagellar Motility Are Affected in l‐Glutamate Overproduction Conditions

To investigate the genetic regulation of *B. methanolicus* MGA3 under l‐glutamate overproduction and export, we aimed to obtain a general overview of transcriptional changes by comparing transcript abundances in cultures grown in MVcM and MVcMii media using RNA sequencing. Altogether, 51 genes were upregulated and 35 genes downregulated in *B. methanolicus* MGA3 under growth in MVcMii in comparison to MVcM (Tables 2, 3, 4, 5). Most upregulated genes are concentrated within 10 distinct genome clusters, involved in metabolic processes such as sporulation, type II secretion, phosphate transport, as well as mannitol catabolism and pyrimidine biosynthesis (Table 2). Indeed, the upregulation of pyrimidine biosynthetic genes was previously observed in *B. methanolicus* mutant strains that overproduce l‐glutamate (Irla et al. 2025).

**TABLE 2:** List of upregulated genes (*n* = 51) in *B. methanolicus* MGA3 cultivated in MVcMii in comparison to MVcM medium.

Gene name	Locus tag	Gene function	Log_2_ FC*
*spoIIE*	BMMGA3_00365	Stage II sporulation protein E	0.77
*yabS*	BMMGA3_00370	Putative protein YabS	0.77
*yabT*	BMMGA3_00375	Putative serine/threonine‐protein kinase YabT	0.85
*mtlA*	BMMGA3_01060	PTS system mannitol‐specific EIICB component	5.73
*mtlR*	BMMGA3_01065	Transcriptional regulator MtlR	4.98
*mtlF*	BMMGA3_01070	Mannitol‐specific phosphotransferase enzyme IIA component	4.88
*mtlD*	BMMGA3_01075	Mannitol‐1‐phosphate 5‐dehydrogenase	5.00
BMMGA3_01500	BMMGA3_01500	CpaF family protein	0.98
BMMGA3_01505	BMMGA3_01505	Type II secretion system F family protein	0.77
BMMGA3_01510	BMMGA3_01510	Type II secretion system F family protein	0.78
*pyrR*	BMMGA3_05780	Bifunctional protein PyrR	1.65
*pyrP*	BMMGA3_05785	Uracil permease	3.04
*pyrB*	BMMGA3_05790	Aspartate carbamoyltransferase	3.86
*pyrC*	BMMGA3_05795	Dihydroorotase	4.26
*pyrAA*	BMMGA3_05800	Carbamoyl‐phosphate synthase pyrimidine‐specific small chain	4.40
*pyrAB*	BMMGA3_05805	Carbamoyl‐phosphate synthase pyrimidine‐specific large chain	4.69
*pyrK*	BMMGA3_05810	Dihydroorotate dehydrogenase B NAD^+^, electron transfer subunit	4.77
*pyrD*	BMMGA3_05815	Dihydroorotate dehydrogenase B NAD^+^, catalytic subunit	5.03
*pyrF*	BMMGA3_05820	Orotidine 5′‐phosphate decarboxylase	4.70
*pyrE*	BMMGA3_05825	Orotate phosphoribosyltransferase	3.41
*mreBH*	BMMGA3_06550	Protein MreBH	0.89
BMMGA3_06955	BMMGA3_06955	Putative membrane protein	1.18
BMMGA3_08230	BMMGA3_08230	MerR family transcriptional regulator	2.00
BMMGA3_08235	BMMGA3_08235	YocH cell wall binding protein	1.00
BMMGA3_11730	BMMGA3_11730	phosphate transport system regulatory protein PhoU	0.89
*pstB*	BMMGA3_11735	Phosphate import ATP‐binding protein PstB	0.98
BMMGA3_as0066	BMMGA3_as0066	Antisense transcript	1.08
BMMGA3_as0067	BMMGA3_as0067	Antisense transcript	1.05
BMMGA3_r00055	BMMGA3_r00055	23S ribosomal RNA	1.31
BMMGA3_r00160	BMMGA3_r00160	23S ribosomal RNA	1.33
BMMGA3_r00465	BMMGA3_r00465	23S ribosomal RNA	1.30
BMMGA3_r00525	BMMGA3_r00525	23S ribosomal RNA	1.25
BMMGA3_r00910	BMMGA3_r00910	16S ribosomal RNA	1.70
BMMGA3_r00915	BMMGA3_r00915	23S ribosomal RNA	1.22
BMMGA3_r01415	BMMGA3_r01415	23S ribosomal RNA	1.15
BMMGA3_r01620	BMMGA3_r01620	23S ribosomal RNA	1.30
BMMGA3_r03535	BMMGA3_r03535	23S ribosomal RNA	1.36
BMMGA3_r13855	BMMGA3_r13855	23S ribosomal RNA	1.26
BMMGA3_r13860	BMMGA3_r13860	16S ribosomal RNA	1.69
BMMGA3_t03615	BMMGA3_t03615	tRNA‐Leu	0.92
BMMGA3_t03620	BMMGA3_t03620	tRNA‐Leu	0.92
BMMGA3_01530	BMMGA3_01530	Hypothetical protein	0.82
*isp*	BMMGA3_01825	Intracellular serine protease	0.72
BMMGA3_02035	BMMGA3_02035	Putative secreted protein	1.50
*yqgI*	BMMGA3_02905	Putative ABC transporter permease protein YqgI	1.01
*des*	BMMGA3_08030	Fatty acid desaturase	0.77
*deoB*	BMMGA3_11100	Phosphopentomutase	0.94
*gerM*	BMMGA3_12800	Spore germination protein GerM	1.14
BMMGA3_16525	BMMGA3_16525	ABC transporter	0.83
*ssrS2*	BMMGA3_s0220	6S RNA	0.97
ssrA	BMMGA3_s0230	Transfer‐messenger RNA	1.31

* Log2 fold change values (*p* ≤ 0.01). List ordered by genomic position of gene clusters, genes in grey are not clustered in *B. methanolicus* genome.

The upregulation of the genes involved in mannitol catabolism was expected as an intrinsic response to the carbon source introduced in the MVcMii medium composition. The presence of mannitol led to upregulation of the operon *mtlARFD* (Table 2), essential for mannitol uptake and catabolism in MGA3 (Heggeset et al. 2012). This operon is also upregulated when mannitol is utilised as the carbon source, compared to when arabitol is used (López et al. 2019). Furthermore, beyond its commercial interest regarding l‐glutamate production, the l‐glutamate biosynthesis pathway is crucial for nitrogen assimilation and serves as a building block provider for several essential compounds in living cells. Among these essential compounds, l‐glutamate plays a major role in supplying nitrogen atoms in the biosynthetic pathway of pyrimidine and purine ribonucleotides (Walker and van der Donk 2016; Kilstrup et al. 2005). Accordingly, genes within the pyrimidine nucleotide biosynthesis operon (*n* = 10) were upregulated in response to l‐glutamate overproduction (i.e., under growth in MVcMii) (Table 2). Not clustered with pyrimidine salvaging genes, a phosphopentomutase (*deoB*), involved in salvaging and interconversion of these nucleotides, was also upregulated (Table 2). Similarly, those genes were upregulated in l‐glutamate‐overproducing mutant strains of *B. methanolicus* (Irla et al. 2025).

Additionally, under MVcMii conditions, we observed downregulation of gene clusters associated with metabolic processes related to cell projection, belonging to phosphotransferase systems (PTS), and arabitol metabolism (Table 3). Arabitol, like mannitol, is a sugar alcohol (pentitol) biosynthesised as a reduction product from sugars such as arabinose and xylose in microorganisms (Ravikumar et al. 2022). This compound was identified as a carbon source capable of supporting the growth of *B. methanolicus* MGA3 (López et al. 2019). In this study, the *atlABCD* gene cluster, responsible for arabitol uptake and its conversion to xylulose 5‐phosphate in *B. methanolicus* , was significantly downregulated under MVcMii conditions (Table 3). This finding agrees with observations by López et al., who reported similar downregulation of this gene cluster under mannitol conditions. When comparing growth on mannitol and arabitol as carbon sources, they found that growth on mannitol led to the downregulation of those genes which were later characterised as associated with arabitol catabolism in *B. methanolicus* (López et al. 2019). Therefore, the findings of the present study corroborate previous research regarding the effects of mannitol supplementation in the *B. methanolicus* growth media.

**TABLE 3:** List of downregulated genes (*n* = 35) in *B. methanolicus* MGA3 cultivated in MVcMii in comparison to MVcM medium.

Gene name	Locus tag	Gene function	Log_2_ FC*
BMMGA3_07550	BMMGA3_07550	Transcriptional antiterminator BglG	−3.28
*atlA*	BMMGA3_07555	IIA arabitol PTS component	−3.34
*atlB*	BMMGA3_07560	IIB arabitol PTS component	−3.03
*atlC*	BMMGA3_07565	IIC arabitol PTS component	−2.59
*atlD*	BMMGA3_07570	Arabitol phosphate dehydrogenase	−2.97
BMMGA3_07575	BMMGA3_07575	Hypothetical protein	−2.65
BMMGA3_07580	BMMGA3_07580	Galatiol‐1‐phosphate dehydrogenase	−1.04
BMMGA3_14950	BMMGA3_14950	Flagellar protein FliT	−1.29
BMMGA3_14955	BMMGA3_14955	Flagellar protein FliS	−1.44
*fliD*	BMMGA3_14960	Flagellar capping protein	−1.55
BMMGA3_14965	BMMGA3_14965	Flagellar protein FlaG	−1.89
BMMGA3_02575	BMMGA3_02575	DUF3906 family protein	−0.88
BMMGA3_02830	BMMGA3_02830	Glutamine ABC transporter periplasmic‐binding component	−0.81
*motA*	BMMGA3_03240	Motility protein A	−1.65
BMMGA3_03885	BMMGA3_03885	MerR family transcriptional regulator	−0.76
*fabH*	BMMGA3_04490	3‐Oxoacyl‐[acyl‐carrier‐protein] synthase 3	−1.14
BMMGA3_04725	BMMGA3_04725	Methyl‐accepting chemotaxis sensory transducer	−2.27
BMMGA3_04730	BMMGA3_04730	Amino acid adenylation domain protein	−1.62
*flgB*	BMMGA3_06100	Flagellar basal body rod protein FlgB	−0.89
BMMGA3_06875	BMMGA3_06875	IDEAL domain‐containing protein	−0.81
BMMGA3_07115	BMMGA3_07115	Hypothetical protein	−1.86
BMMGA3_07145	BMMGA3_07145	Spore coat protein	−2.31
BMMGA3_07290	BMMGA3_07290	Competence protein CoiA‐like family	−0.82
BMMGA3_08510	BMMGA3_08510	Anti‐repressor SinI family protein	−0.94
BMMGA3_09580	BMMGA3_09580	Putative membrane protein	−1.98
BMMGA3_12590	BMMGA3_12590	Type II secretion system GspH family protein	−0.94
BMMGA3_12620	BMMGA3_12620	DUF4229 domain‐containing protein	−0.95
*fadR*	BMMGA3_12875	Fatty acid metabolism regulator protein	−0.90
BMMGA3_14285	BMMGA3_14285	Branched chain amino acid ABC transporter	−0.87
BMMGA3_14975	BMMGA3_14975	Flagellin domain‐containing protein	−1.88
BMMGA3_15620	BMMGA3_15620	Helix‐turn‐helix domain‐containing protein	−1.84
BMMGA3_15635	BMMGA3_15635	SpoIID/LytB domain‐containing protein	−0.85
*mutB2*	BMMGA3_16155	Methylmalonyl‐CoA mutase, large subunit	−1.20
*acdA*	BMMGA3_16165	Acyl‐CoA dehydrogenase	−1.96
*fadF*	BMMGA3_16185	Putative protein FadF	−1.21

* Log_2_ fold change values (*p* ≤ 0.01). List ordered by genomic position of gene clusters, genes in grey are not clustered in *B. methanolicus* genome.

The downregulation of cell projection genes (clustered in the genome: *fliTSD* and *flaG*) under MVcMii conditions (Table 3) may be linked to the metabolic investment in l‐glutamate overproduction by MGA3. Flagellar motility and chemotaxis are known to be highly energy‐demanding processes; in *Pseudomonas syringae* , for instance, flagellar biosynthesis and function consume around 2% of the total cellular energy (Hockett et al. 2013). Moreover, conserving energy is recognised as a strategy that allows bacteria to allocate resources to other metabolic processes. For instance, during biofilm formation, bacteria downregulate the expression of the gene encoding the FliD capping protein, helping to conserve energy and support adherence (Haiko and Westerlund‐Wikström 2013). Indeed, l‐glutamate overproducing mutants of *B. methanolicus* promote antagonistic regulation between cell projection and l‐glutamate production (Irla et al. 2025).

### 2.3 Fatty Acids Metabolism is Involved in l‐Glutamate Overproduction

**FIGURE 1:**
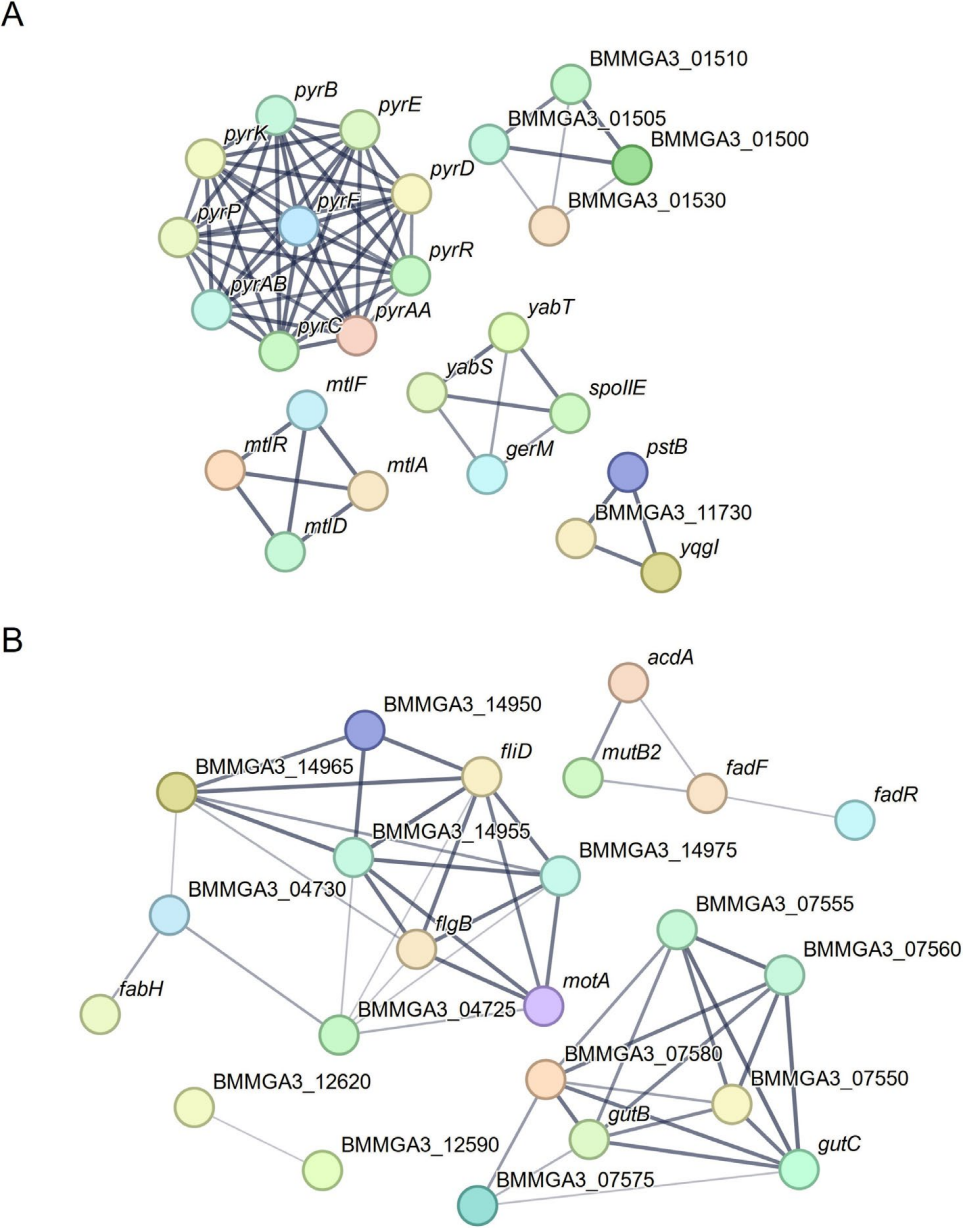
Interaction network of proteins coded by genes upregulated (A) and downregulated (B) in *B. methanolicus* MGA3 cultivated in MVcMii in comparison to MVcM medium. Protein–protein interaction network analysis (interaction score of 0.4) was done using the STRING database (version 12.0). The edges indicate functional and physical protein associations; line thickness indicates the strength of data support. Downregulation of fatty acid biosynthesis genes may indirectly activate MscS by altering membrane fluidity.

To gain a deeper understanding of the processes involving differentially expressed genes, especially those not clustered in the genome, we conducted *in silico* protein–protein interaction analysis grouping functionally and physically similar genes. This analysis evidenced grouped genes upregulated under MVcMii conditions within pyrimidine biosynthesis, mannitol catabolism, phosphate transfer, and spore formation processes (Figure 1A). In contrast, among the downregulated genes, aside from the clustered arabitol catabolism and cell projection, we observed genes associated with fatty acid biosynthesis (Figure 1B). Specifically, *fadR*, *fadF*, *mutB2*, and *acdA* encode the fatty acid metabolism regulator protein, putative protein FadF, methylmalonyl‐CoA mutase (large subunit), and acyl‐CoA dehydrogenase, respectively. Those genes are grouped within the STRING term CL:1622, indicating a mixed category including “carbon metabolism and carbohydrate transport” with a false discovery rate (FDR) of 7.45e‐05 (data not shown). Similarly, l‐glutamate overproducing mutants of *B. methanolicus* presented mutations in fatty acids biosynthesis genes *fabI* and *fabG*, encoding an enoyl‐[acyl‐carrier‐protein] reductase [NADH] and a malonyl‐CoA acyl carrier protein transacylase, respectively (Irla et al. 2025). In the same study, transcriptomic comparison between mutants and wild‐type revealed coordinated changes in genes involved in fatty acid and glycerophospholipid metabolism. Notably, genes encoding enzymes for glycerophospholipid synthesis were downregulated, while genes linked to membrane lipid and fatty acid metabolism were upregulated. Those metabolic shifts likely contribute to structural changes in the cell membrane under l‐glutamate overproduction (Irla et al. 2025). In *Bacillus subtilis* and *E. coli* , genes for fatty acid biosynthesis are also scattered throughout the chromosome (Parsons and Rock 2013). Furthermore, the downregulation of *fadR* might be crucial to understanding the role of the biosynthesis of fatty acids in the l‐glutamate overproduction by *B. methanolicus* observed in this study (Table 1). The FadR protein in *B. methanolicus* (RefSeq: WP_003347520.1) exhibits an 82.29% identity with a TetR/AcR family transcription regulator found in Bacillaceae (data not shown). However, this regulator has not been previously characterised. Homologues of the *E. coli* FadR are exclusively found in a subset of γ‐proteobacteria that colonise animals or plants, suggesting the involvement of FadR regulation in the utilisation of fatty acids within these ecological niches (Iram and Cronan 2005). In *B. subtilis* , a transcriptional regulator named FadR represses genes related to β‐oxidation but does not serve as an activator for genes involved in fatty acid biosynthesis. Notably, *B. subtilis* and *E. coli* FadR proteins exhibit structural differences; *B. subtilis* FadR belongs to the TetR family, whereas *E. coli* FadR is a GntR‐like repressor (Fujita et al. 2007).

Interestingly, one fatty acid desaturase (*des*) was upregulated under MVcMii conditions (Table 2). This enzyme family catalyses the conversion of saturated fatty acids into unsaturated and polyunsaturated forms, which has important implications for l‐glutamate export. The cell membrane contains lipids whose hydrophobic moieties are linear saturated and unsaturated fatty acids. Bacteria can modify their membrane lipid composition in response to environmental changes. For example, the balance between saturated and unsaturated fatty acids in *E. coli* and other bacteria changes to maintain appropriate membrane fluidity (i.e., the viscosity of the lipid bilayer reflecting how lipids and proteins move laterally within the membrane plane) at different temperatures (Sohlenkamp and Geiger 2016). In the context of this study, we hypothesize that in *B. methanolicus* MGA3, an increased proportion of unsaturated fatty acids may alter membrane properties such as fluidity or tension, which in turn could modulate the activity of mechanosensitive channels and influence l‐glutamate efflux (Nakayama, Hashimoto, et al. 2018). This proposed mechanism is consistent with how lipids contribute to the gating of mechanosensitive channels in *E. coli* , where an increasing degree of unsaturated fatty acids leads to the opening of large‐conductance mechanosensitive channels (MscL) and stabilises the open conformation of MscS (Flegler et al. 2022).

Additionally, an ungrouped 3‐oxoacyl‐[acyl‐carrier‐protein] synthase gene *fabH*, associated with the fatty acid biosynthesis pathway, was identified below the established FDR for protein interaction (Table 3). In *E. coli* , the fatty acid biosynthesis is initiated by FabH, which performs the condensation of malonyl‐acetyl carrier protein (ACP) with acetyl‐CoA, initiating the fatty acid elongation cycle (López‐Lara and Soto 2019). Membrane fluidity in bacteria is closely related to metabolic pathways for lipids and fatty acids biosynthesis. Generally, Firmicutes, except for lactobacteria, possess branched‐chain fatty acids (i.e., iso−/anteiso‐) in their lipid structure. Membranes are formed by amphiphilic lipids, mostly glycerophospholipids. They consist of a glycerol moiety, two fatty acids, and a phosphate group with a variable head group. Examples are phosphatidylethanolamine, phosphatidylglycerol, cardiolipin, lysyl‐phosphatidylglycerol, phosphatidylinositol, phosphatidic acid, and phosphatidylserine. Bacteria can also form phosphorus‐free membrane lipids such as ornithine lipids, sulfolipids, diacylglyceryl‐*N*,*N*,*N*‐trimethylhomoserine, glycolipids, diacylglycerol, hopanoids, and others (Sohlenkamp and Geiger 2016). Our RNA sequencing analysis revealed downregulation of glutamine and branched‐chain (BC) amino acid transporters (Table 3). In bacteria like *B. subtilis* , BC fatty acids are the major constituents of membrane lipids. These are synthesised by incorporating a BC acyl‐CoA, e.g., methylmalonyl‐CoA, as a precursor, which is elongated through the fatty acid synthase pathway (Butterworth and Bloch 1970). Hence, our finding is significant because branched‐chain amino acids serve as precursors for methylmalonyl‐CoA synthesis, influencing BC fatty acid and lipid production, ultimately affecting membrane assembly (Beck 2005). Overall, these results suggest that *B. methanolicus* MGA3 modifies its membrane composition to facilitate l‐glutamate efflux through mechanosensitive channels.

### 2.4 MscS From *B. methanolicus* Resembles That of *E. coli* and Displays Sequence Indicators of Slow Gating

Here, we employed *in silico* analyses of the *B*. *methanolicus* MscS aiming to confirm its small‐conductance mechanosensitive channel structure. In MscS‐like transporters involved in metabolite export, such as MscCG and MscCG2 in *C. glutamicum* , slower channel opening kinetics, compared to the non‐specific *E. coli* MscS, are considered necessary for an effective response to metabolite‐induced stress (Nakayama et al. 2013). A multiple‐sequence alignment shows that the amino acid sequence of MscS in *B. methanolicus* MGA3 (UniProt: I3EBZ0) shares identity with *E. coli* MscS (UniProt: P0C0S1) and *C. glutamicum* MscCG (UniProt: P42531), of 25.8% and 30.8%, respectively (Figure S1). The sequence logo presented in Figure S1 further highlights the conservation of hydrophobic patches within the first TM helix across highly diverse MscS sequences, suggesting structural features crucial for function. Notably, within the genome of *B. methanolicus* MGA3, only a singular gene annotated as small‐conductance mechanosensitive channel *mscS* (BMMGA3_16700) is present. However, there is an absence of genes encoding homologues of MscL (data not shown).

Furthermore, mutations at positions G113 and N117 in the MscS transporter of *E. coli* have been shown to reduce transporter activity (Rowe et al. 2014; Akitake et al. 2007). The MscS‐like transporter in *B. methanolicus* lacks the typical extended C‐terminus found in MscCG transporters (Figure S2), which is involved in slowing down opening kinetics (Nakayama et al. 2016). However, specific residues in both MscCG and MscCG2 have also been associated with this property, since MscCG2, despite lacking an extended C‐terminus, still exhibits slower opening kinetics than *E. coli* MscS. Notably, two mutations related to the *E. coli* transporter, G113S and N117D, have been identified, while other key residues remain conserved (Nakayama, Komazawa, et al. 2018). Here, the newly identified MscS‐like transporter in *B. methanolicus* exhibits the same mutations as the *C. glutamicum* transporter MscCG2, as well as a conserved sequence in the vicinity of these positions, suggesting a similar functional behavior (Figure S3). Hence, sequence comparison with *C. glutamicum* MscCG and MscCG2 indicates that *B. methanolicus* channel dynamics partially resemble those of *C. glutamicum* , with similar mutations likely leading to slow channel opening kinetics and enabling metabolic coupling.

In the plasma membrane of *E. coli* , both MscS and MscL demonstrate an interplay between lipid properties and protein function, acting redundantly to prevent cell lysis during hypoosmotic shock (Schumann et al. 2010). Bacterial and archaeal genomes often host MscS‐encoding genes, with *E. coli* possessing five such genes (Booth et al. 2011). Homologues of MscL and MscS have been shown to be essential for the survival of hypoosmotic shock in *B. subtilis* during logarithmic phase (Wahome and Setlow 2008; Wahome et al. 2009). Hence, it has been suggested that this function is conserved in Gram‐positive bacteria (Haswell et al. 2011). The *mscS*‐like gene *NCgl1221* has been historically established as responsible for l‐glutamate excretion in *C. glutamicum* (Hashimoto et al. 2012; Nakamura et al. 2007; Yao et al. 2009). Later, in the *C. glutamicum* genome, single homologues were found for each of *E. coli* 's MscS and MscL (Nottebrock et al. 2003). Like in *E. coli* , they act as osmotic safety valves, regulating cellular turgor pressure during hypoosmotic conditions. Interestingly, the opening of the *C. glutamicum* mechanosensitive channel MscCG primarily enables the efflux of l‐glutamate through a “force‐from‐lipids” gating mechanism (Nakayama, Hashimoto, et al. 2018).

**FIGURE 2:**
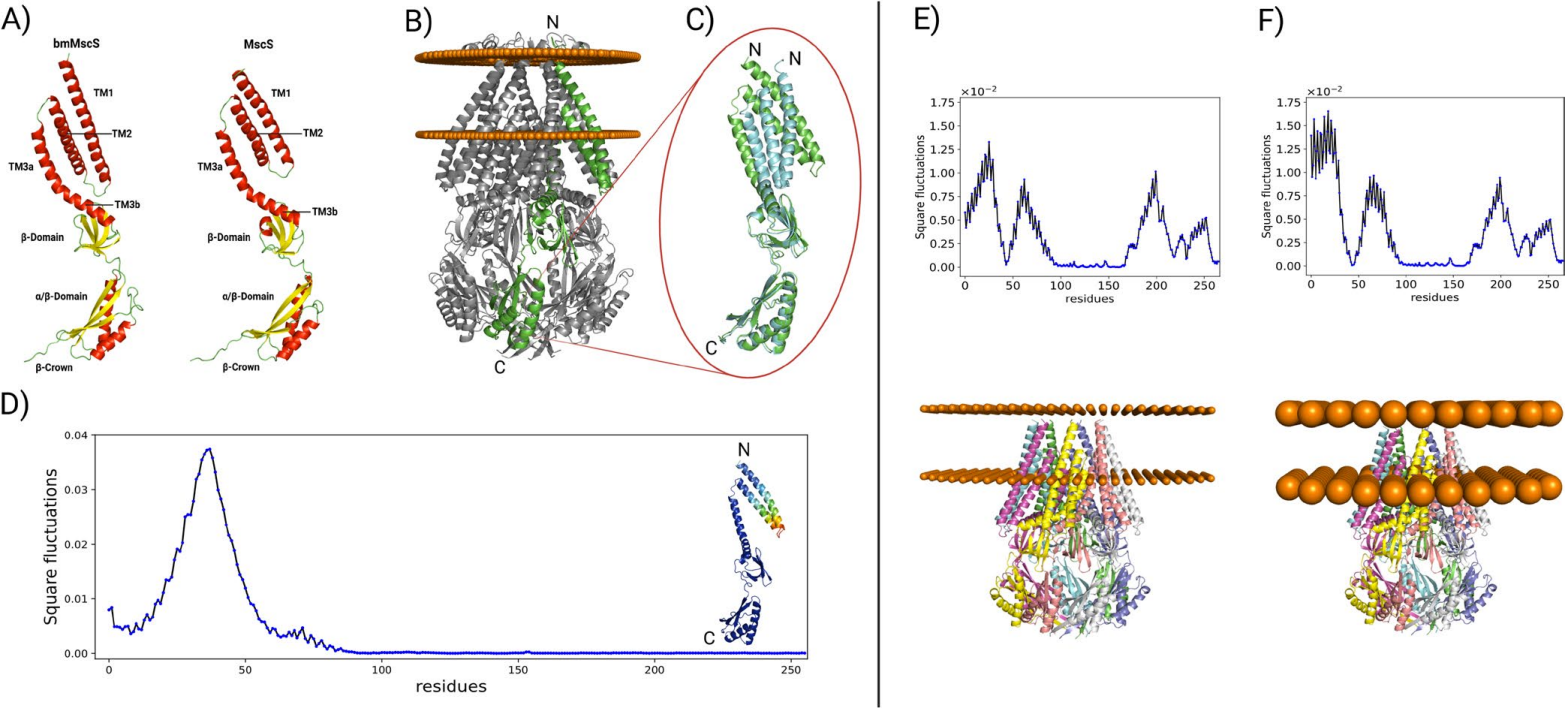
(A) Comparison between the monomer structure of *B. subtilis* MGA3 (bmMscS; left) and *E. coli* MscS (right), highlighting their high structural similarity, characterised by three transmembrane α‐helices and conserved cytoplasmic domains. (B) Crystal structure of *E. coli* MscS transporter in closed conformation [PDB 6PWN] oriented in a phospholipid bilayer predicted by OPM. One monomer of the homoheptamer is highlighted in green. (C) Superimposition of the monomers in the open [PDB 2VV5] and closed conformation [PDB 6PWN] of *E. coli* MscS transporter. (D) The main motion is the rotation of the helices TM1, TM2 and TM3a as shown by the square fluctuation of the normalised deformation vector which is colour coded in the monomer structure on the right side. (E) In the top panel the square displacement of the first normal mode for one monomer which shows the displacement of the TM region and the soluble domain. In the bottom panel, the MGA3 transporter AF3 predicted structure with short termini embedded in the membrane modelled as an elastic network with a node radius of 3.1. The monomers of the homoheptamer are highlighted with different colours. (F) The top panel shows how the square fluctuations change in this membrane. In the bottom panel, the MGA3 transporter is embedded in an elastic network with bigger nodes (radius 7.0).

To further explore the structural organisation of MscS as a mechanosensitive channel, its structure was predicted using the AlphaFold 3 webserver (AF3, https://alphafoldserver.com/). AF3 does not only predict the structure of a monomer, but also the interaction between monomers to build up multimeric protein structures. In spite of their low sequence identity, the *B. methanolicus* MGA3‐derived MscS *in silico* 3D structure resembles that of *E. coli* (Kawasaki and Martinac 2020). The comparison between the monomers of *E. coli* MscS and *B. methanolicus* MGA3 reveals a high degree of structural similarity (Figure 2A). The transmembrane domain consists of three α‐helices, named TM1, TM2, and TM3a/TM3b (Figure 2A). The cytoplasmic region is organised into a β‐domain, an α/β‐domain, and a β‐crown, a structural arrangement also observed in the *E. coli* MscS monomer. As in other MscS‐like transporters, the monomers assemble into the characteristic homoheptameric complex (Figure S2). By comparing the transmembrane region conformation of the predicted structure and the reference MscS transporter from *E. coli* , our obtained structure is in the closed conformation, but the helices TM1 and TM2 are slightly longer than the reference protein.

The interaction with a model membrane was predicted by using the online server OPM (Lomize et al. 2022), which shows how the protein is oriented into a Gram‐positive membrane. The OPM server predicted that the MscS‐membrane complexes in *B. methanolicus* MGA3 and *E. coli* are similar (Figure 2B, Figure S2 and Table S1). From the predicted structure of MGA3 MscS, it is also possible to determine the localisation of the hook lipids that are known to play an important role in the molecular events involved in the opening motion (Kawasaki and Martinac 2020). In the crystal structure of the closed *E. coli* MscS transporter (PDB: 6PWN), the hook lipids are localised in their binding pocket by interacting with the positively charged residue R88 and residue Y27 (Reddy et al. 2019). Interestingly, in the same structural positions of the predicted MGA3 MscS structure, a similar chemical environment can be observed with a positive residue K33 and two hydrogen bond donor residues S99 and T29, which could be a possible binding region for the hook lipids.

The electrostatic map (Figure S2) inside the channel shows walls rich in lysine residues, which create a positive patch consistent with a channel whose role is to transport a negatively charged molecule like l‐glutamate. We analyzed the opening mechanism of the MscS channel in *E. coli* by comparing the crystal structure of the open and closed conformations (Wang et al. 2008; Lezon and Bahar 2012). This structural change involves synchronised rotation and flipping of the transmembrane helices (Figure 2C). This motion reorients the side chains of the phenylalanine residues localised in the hole of the channel, leading to the opening of the pore. In Figure 2, this key conformational change is represented by the normalised squared fluctuations of Cα in one monomer of the homoheptamer. It can be clearly seen that the extramembrane portion of the monomer is not affected by any motion, while the transmembrane region experiences a displacement of the backbone that is described by a Gaussian‐like distribution (Figure 2D). Given the structural similarity between the reference *E. coli* protein and the MGA3 channel, we hypothesize that the opening mechanism of MGA3 MscS exhibits similar dynamics.

To investigate how the membrane influences the dynamics of the channel, we modeled the MGA3 MscS structure as an elastic network where each residue is represented by a node centered at Cα and connected to other nodes via springs. Similarly, the membrane was modeled as an elastic network in which nodes represent groups of lipid molecules, resembling the interactions within a phospholipid bilayer (Lezon and Bahar 2012). Representing the protein‐membrane system as two elastic networks allowed us to rapidly assess the dynamics of the protein through normal mode analysis (NMA), which is a fast computational method to gain insights into the backbone dynamics of proteins. The elementary motions evaluated using NMA are ranked based on their frequency, with the highest ranked motion being the slowest one, and therefore most biologically relevant (Doruker et al. 2000). The squared fluctuations of the slowest motion for one monomer of MGA3 MscS are reported in Figure 2E. This motion is characterised by a rotation of the TM α‐helices concurrent with the anti‐rotation of the cytoplasmic domain. Notably, there is a similarity in the opening motion of the TM α‐helices in both the MGA3 and *E. coli* proteins. However, NMA does not fully capture the opening dynamics of MscS, as this motion also involves side‐chain dynamics, which are not considered in the model. Despite this limitation, the slowest motion of MGA3 MscS still captures a key aspect of membrane dynamics related to the opening and closing of the channel, namely the rotation of the helices.

We investigated the influence of membrane properties on the dynamics of MGA3 MscS to address the extent to which the channel may be affected by mechanical signals from the membrane. This was achieved by increasing the size of the membrane nodes. Since the volume of the membrane is kept constant, this effectively reduces the density of phospholipids in the elastic network model of the membrane (Figure 2F). NMA of MGA3 MscS embedded in the lower density membrane revealed changes in the dynamics of the slowest motion. The rotation speed of the TM helices became faster than the cytoplasmic region and it involved a larger portion of the helices (Figure 2F). This suggests that changing the properties of the membrane influences the opening dynamics of MGA3 MscS (Lezon and Bahar 2012). This model strengthens the regulatory response to l‐glutamate overproduction observed in this study, where upregulation of fatty acid desaturases was observed under MVcMii conditions, indicating the formation of unsaturated fatty acids (Table 2). Hence, variations in lipid density or composition within the MGA3 membrane are likely to trigger the efflux of l‐glutamate through MscS. *B. methanolicus* MGA3 may adopt this mechanism when the membrane is stretched, facilitating the opening of its mechanosensitive channel MscS. Future studies should experimentally investigate the functional role of the identified amino acid residues in MscS, for example through site‐directed mutagenesis and analysis of l‐glutamate export. Such work would help validate the *in silico* predictions and provide deeper insight into the molecular mechanism of MscS‐mediated l‐glutamate efflux in *B. methanolicus* .

### 2.5 Increase in Biotin Supply Reduce l‐Glutamate Production in *B. methanolicus*

Next, we evaluated l‐glutamate production under conditions of induced membrane tension in vivo. Research conducted on *C. glutamicum* by Hoischen and Krämer in 1990 indicates that under biotin limitation conditions, the total amount of membrane lipids in *C. glutamicum* is nearly halved, and the ratio of saturated to unsaturated lipids (predominantly palmitic acid (C16:0) and oleic acid (C18:1)) in the membrane increases. They suggested the potential existence of a carrier‐mediated l‐glutamate secretion system that responds to alterations in lipid environments induced by biotin limitation (Hoischen and Krämer 1990).

**FIGURE 3:**
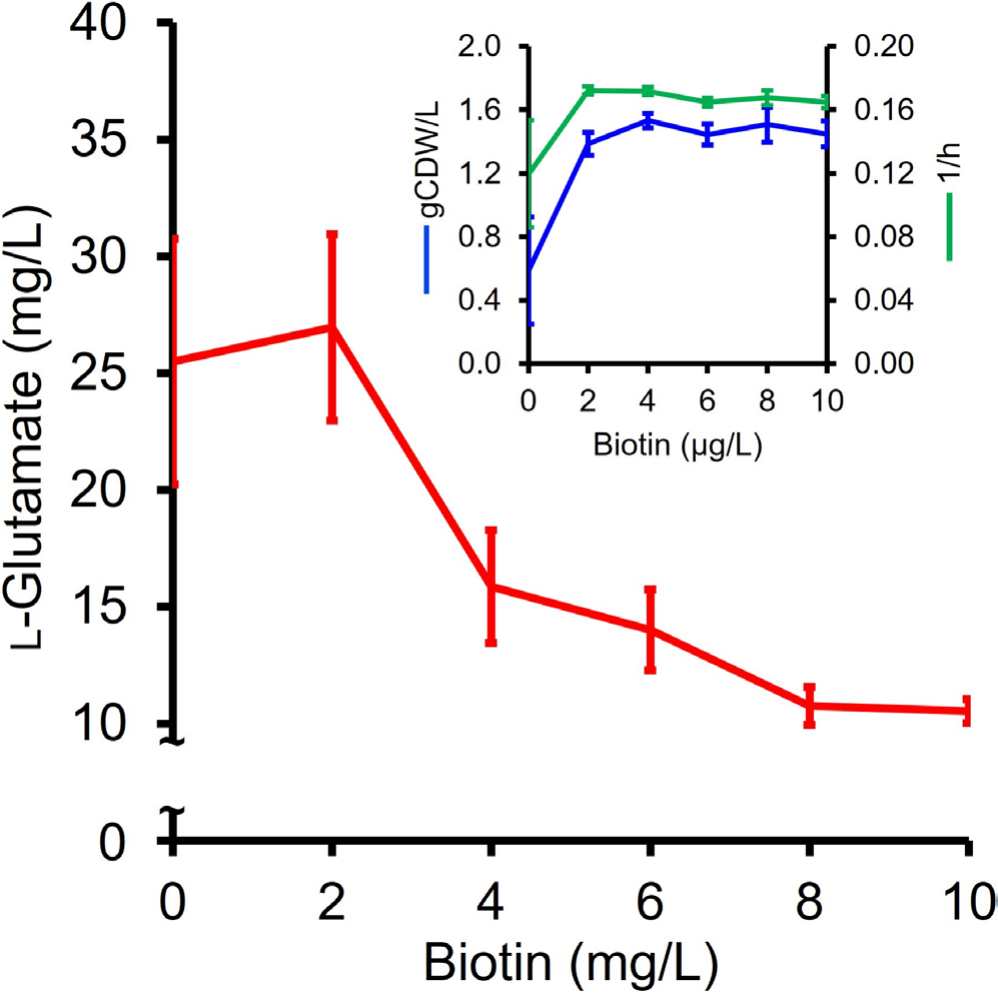
Effect of biotin supplementation on growth and l‐glutamate production in *B. methanolicus* MGA3. Biotin‐supplied *B. methanolicus* MGA3 cells were cultivated in MVcM medium in Duetz system plates, and the cultures were supplemented with 0.1, 2, 4, 6, 8, or 10 mg/L biotin. The growth was monitored, culture supernatants were taken after 24 h, and their l‐glutamate content was quantified by means of HPLC. Lines and error bars represent means and standard deviations of technical triplicates.

In lactic acid bacteria, the supplementation of Tween 80 to the growth media downregulates endogenous fatty acid biosynthesis and boosts levels of unsaturated fatty acids, oleic acid and cyclopropane (Reitermayer et al. 2018). More recent work with multiple *Lactobacillus* strains showed that Tween 80 alters membrane fluidity by reshaping the balance of saturated vs. unsaturated fatty acids (Zaręba and Ziarno 2024). Schendel et al. previously demonstrated that adding surfactants to the growth media increased l‐glutamate export. Specifically, supplementing the media with 2 g/L Tween 80 led to a 2.5‐fold increase in l‐glutamate titre compared to controls without Tween in *B. methanolicus* MGA3. Together, these findings strongly suggest that surfactants may facilitate l‐glutamate export by altering membrane properties in this organism. However, varying conditions, such as biotin limitation and the addition of beta‐lactam antibiotics like penicillin, negatively impact biomass formation. This, in turn, hampers l‐glutamate production by disrupting cell growth and metabolic activity in *B. methanolicus* (Schendel et al. 2000). In this study, the wild‐type strain MGA3 was subjected to a gradual increase in biotin supply. Notably, *B. methanolicus* relies on biotin and cyanocobalamin supplementation for growth. Therefore, the MVcM medium was modified by supplementing it with only 0.01 mg/L cyanocobalamin and varying biotin concentrations (0.1, 2, 4, 6, 8, or 10 mg/L) instead of the standard vitamin mixture described in Table 5. The biotin levels ranging from 2 to 10 mg/L represent a 20 to 1000‐fold increase compared to the commonly used 0.1 mg/L biotin concentration in the standard MVcM medium. Across the range of biotin concentrations from 2 to 10 mg/L, there were no discernible effects on the final biomass (g CDW/L) and growth rates (1/h) of MGA3. However, under 0.1 mg/L biotin supplementation, a substantial impact was observed. The final biomass decreased by 43%, and the cell growth rate slowed by 50% (Figure 3). Biotin supply significantly influenced l‐glutamate production in MGA3. Supplementation of 0.1 mg/L and 2 mg/L biotin resulted in 25 ± 5 mg/L and 27 ± 4 mg/L l‐glutamate, respectively, despite the observed poor growth in the former condition. Notably, the disparity in l‐glutamate production observed under the control condition with 0.1 mg/L biotin supplementation (Figure 3) vs. the MVcM medium condition (Table 1) is attributed to differences in cultivation scale; specifically, the small‐scale Duetz well plate format compared to flask‐based conditions used in the latter. As expected, a further increase in biotin supplementation had a detrimental effect on l‐glutamate production in MGA3, leading to approximately a 50% decrease as biotin levels increased (Figure 3). Notably, l‐glutamate production exhibited a plateau from 8 mg/L biotin supplementation onwards, stabilising at around 10 mg/L l‐glutamate (Figure 3). This consistent l‐glutamate production persisted under higher biotin concentrations tested in this study, up to 100 mg/L (data not shown).

Biotin‐dependent acyl‐CoA carboxylases play a central role in fatty acid and polyketide biosynthesis. These enzymes are typically multi‐subunit complexes composed of a homodimeric biotin carboxylase, a heterooligomeric carboxyltransferase, and a biotin carboxyl carrier protein (Polyak et al. 2012; Arabolaza et al. 2010). The modular nature of these subunits allows for the carboxylation of short‐chain acyl‐CoA substrates of varying lengths. The resulting carboxylated products serve as key intermediates in the fatty acid synthesis pathway, contributing to the generation of structurally diverse lipid molecules and consequently membrane homeostasis (Janßen and Steinbüchel 2014).

**FIGURE 4:**
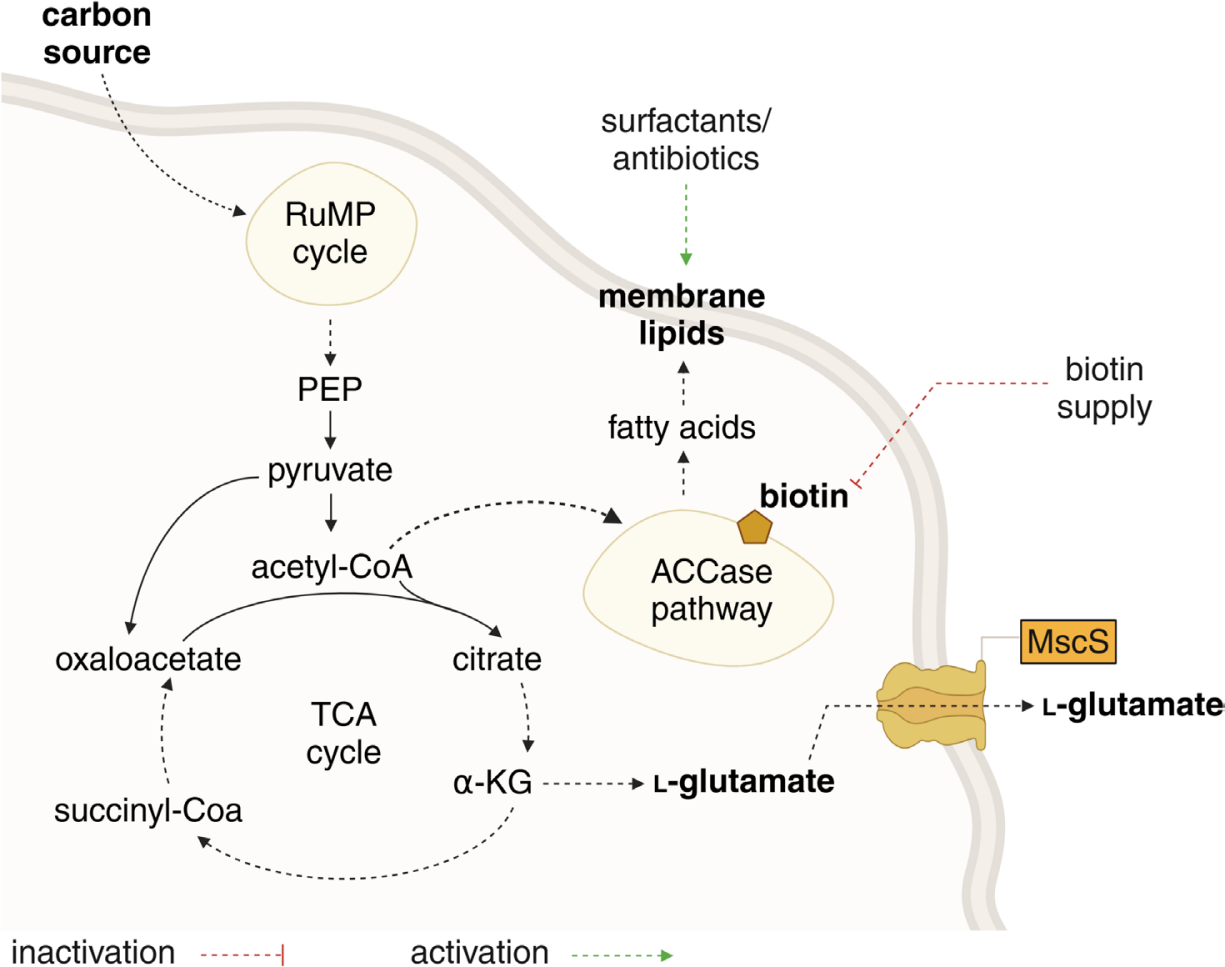
Schematic representation of l‐glutamate biosynthesis in *B. methanolicus* and its putative inactivation/activation export mechanism through a mechanosensitive channel. RuMP, ribulose monophosphate; PEP, phosphoenolpyruvate; TCA, tricarboxylic acid; α‐KG, α‐ketoglutarate; MscS, small‐conductance mechanosensitive channel MscS. Figure created with BioRender (https://BioRender.com/q06v990).

Because *B. methanolicus* cannot synthesize biotin *de novo*, supplementation provides the essential cofactor to the carboxylases involved in fatty acid biosynthesis. Here, enhanced biotin supplementation is expected to upregulate fatty acid biosynthesis, leading to increased lipid production. While this supports membrane integrity and function, it may also divert metabolic precursors such as acetyl‐CoA and energy away from l‐glutamate biosynthesis. l‐Glutamate synthesis in *B. methanolicus* primarily involves the glutamate synthase pathway, which relies on α‐ketoglutarate derived from the TCA cycle (Krog et al. 2013). An increased flux toward fatty acid synthesis could potentially compete for acetyl‐CoA and reduce the availability of α‐ketoglutarate for l‐glutamate biosynthesis (Figure 4), thereby impacting l‐glutamate production efficiency. In contrast, under conditions of l‐glutamate overproduction, *B. methanolicus* exhibits a downregulation of fatty acid biosynthesis (Table 3). This potential trade‐off between lipid and l‐glutamate biosynthesis underscores the need for a balanced metabolic flux to optimise production yields. Further investigations are warranted to elucidate the extent of this metabolic interplay and to develop strategies that can mitigate any negative impacts on l‐glutamate production while maintaining essential lipid synthesis.

### 2.6 Loss‐/Gain‐of‐Function‐Based Analysis Indicate MscS‐Mediated Export of l‐Glutamate in *B. methanolicus*

To further investigate the role of MscS in *B. methanolicus* , we used genetic tools to modulate the expression of the *mscS* gene. Overexpression vs. repression studies demonstrated its influence on cell growth, l‐glutamate efflux, and lipid composition. The strain MGA3(pBV2xp‐*mscs*) represents the overexpression (gain‐of‐function) variant, whereas the strain MGA3(piCas‐*mscS*) represents the repression (loss‐of‐function) variant. Their empty vector counterparts, strains MGA3(pBV2xp) and MGA3(piCas), were used as controls, respectively.

It was previously suggested that MscS might be sensitive to the total membrane lipid content rather than specific lipid components (Reddy et al. 2019). Therefore, the lipid profiling chromatograms performed in this study revealed no apparent changes in the lipid profiles of both gain‐ and loss‐of‐function strains compared to their respective control counterparts (Figure S4). Principal component analysis (PCA) of lipid mass abundances uncovered distinct patterns between the experimental groups. While no significant differentiation was observed between MGA3(pBV2xp) and MGA3(pBV2xp‐*mscS*), a separation was evident between the triplicates of MGA3(piCas) and MGA3(piCas‐*mscS*), with the latter group distinctly clustering apart from MGA3(piCas) (Figure S5). This suggests only a slight divergence in the lipid profiles between these strains. Given the close interplay between mechanosensitive channels and membrane lipid dynamics, it is plausible that repressing the channel triggers compensatory remodelling of the membrane to maintain homeostasis. However, our lipid analysis results suggest that the loss‐ or gain‐of‐function in MscS did not result in major changes in membrane lipid composition in *B. methanolicus* . The impaired l‐glutamate export in the MGA3(piCas‐*mscS*) strain (Table 4) likely adds metabolic and osmotic pressure intracellularly. Since no major alterations in membrane lipid composition were observed upon *mscS* gene repression, the observed membrane‐related responses are more likely due to the metabolic stress imposed by intracellular l‐glutamate accumulation in addition to direct changes in the membrane's physical structure or lipid makeup. Such stress may be explained by the significant growth impair caused by CRISPRi‐mediated repression of the *mscS* gene (Table 4). Intracellular osmotic stress has been documented in *E. coli* strains lacking both MscS and MscL, where impaired solute release leads to markedly reduced survival following hypoosmotic shock (Cox et al. 2013). It is noteworthy that cell death upon *mscS* gene targeting was not observed in our study (Table 4). This may be due to the fact that our CRISPRi system inherently exhibits background expression of the targeted gene, and thus does not achieve complete gene repression (Schultenkämper et al. 2019).

**TABLE 4:** Growth, l‐glutamate production rate and α‐KGDH activity of *B. methanolicus* strains MGA3(pBV22xp) vs. MGA3(pBV2xp‐*mscS*), and MGA3(piCas) vs. MGA3(piCas‐*mscS*). *B. methanolicus* strains were cultivated in MVcM medium (for production assessment) or SOB medium (for α‐KGDH activity assessment) in shake flasks, and the cultures were induced with 1% xylose or 12.5 mannitol at 2 h of growth.

Strain	Growth rate (1/h)	Biomass (g CDW/L)	l‐Glutamate efflux (mg/g CDW/h)	α‐KGDH specific activity (U/mg/min)
MGA3 (pBV2xp)	0.41 ± 0.00*a	1.16 ± 0.06a	9 ± 1b	0.15 ± 0.06a
MGA3 (pBV2xp‐*mscS*)	0.30 ± 0.01b	0.87 ± 0.09b	26 ± 2a	0.20 ± 0.05a
MGA3 (piCas)	0.41 ± 0.03a	1.15 ± 0.09a	42 ± 1a	0.01 ± 0.00a
MGA3 (piCas‐*mscS*)	0.41 ± 0.03a	1.29 ± 0.05a	15 ± 1b	0.02 ± 0.01a

*Note:* Cell culture samples were taken after 3, 5, 7.5 and 21.5 h of growth and their l‐glutamate content was quantified by means of HPLC for l‐glutamate efflux calculation. Cell pellets were harvested 6 h after induction for preparation of crude extracts. The table shows means and standard deviations of technical triplicates. Statistical comparisons were performed between the strain pairs MGA3(pBV2xp) vs. MGA3(pBV2xp‐*mscS*) and MGA3(piCas) vs. MGA3(piCas‐*mscS*). Different letters indicate a significant difference according to the Scott‐Knott test (*p* < 0.05).

* 0.0038.

To further investigate the potential metabolic differences between the strains, we conducted activity assays of α‐ketoglutarate dehydrogenase (α‐KGDH). α‐Ketoglutarate is converted either to succinyl‐CoA by α‐KGDH in the TCA cycle or to l‐glutamate (Figure 4). In *B. methanolicus* , α‐KGDH is encoded by the *odhAB* operon, and it is tightly downregulated under increased l‐glutamate production in this organism (Irla et al. 2025). Overexpression of the *odhAB* operon in *B. methanolicus* has been shown to result in an 8‐fold reduction in l‐glutamate production, confirming that it negatively regulates l‐glutamate synthesis in this organism (Krog et al. 2013). We expected that loss‐/gain‐of‐function of MscS would influence a metabolic pull toward l‐glutamate production and modulate the activity of α‐KGDH. However, in our study, α‐KGDH activity assays under conditions of MscS loss−/gain‐of‐function did not reveal any significant regulation of α‐KGDH during alterations in l‐glutamate biosynthesis (Table 3). A similar lack of regulation was observed in transcriptomic analysis under MvcMii medium (Table 4). Despite this, the recombinant cells exhibited clear changes in l‐glutamate efflux. Under the overexpression of the native mechanosensitive channel gene *mscS*, *B. methanolicus* recombinant cells exhibited a decrease in growth rate and biomass of approximately 33% and 25%, respectively, in comparison to the empty vector control strain (Table 4). Despite the growth disparity between these strains, MGA3(pBV2xp‐*mscS*) demonstrated a 2.2‐fold increase in l‐glutamate efflux, i.e., production rate in mg/g CDW/h, compared to the wild‐type strain without overexpression of *mscS* (Table 4). Accordingly, the media assessment in this study showed a similar reduction in the growth rate of *B. methanolicus* MGA3 during l‐glutamate overproduction (Table 1). In contrast, under *mscS* gene repression targeting using the CRISPRi system, while the growth parameters observed in the empty vector control strain MGA3(piCas) were similar to those in the strain MGA3(piCas‐*mscS*), CRISPRi‐induced repression led to a 2.8‐fold reduction in the l‐glutamate efflux (Table 4). Consistent with our findings, previous studies on the Gram‐positive *C. glutamicum* have demonstrated similar outcomes in function engineering. Gene mutations in the *C. glutamicum* gene NCgl1221, which encodes a MscS, effectively abolish l‐glutamate secretion under biotin‐limiting conditions. However, complementation with the wild‐type NCgl1221 gene restores the l‐glutamate secretion phenotype (Nakamura et al. 2007). Accordingly, a gain‐of‐function mutation in the same *C. glutamicum* gene causes spontaneous l‐glutamate efflux (Nakayama, Hashimoto, et al. 2018). Altogether, our study indicates that both gain‐ and loss‐of‐function alterations in the *mscS* gene proportionally impact l‐glutamate efflux in *B. methanolicus* , suggesting that MscS serves as a key exporter of l‐glutamate in this organism.

It is noteworthy that there is a substantial difference in l‐glutamate efflux between the gain‐ and loss‐of‐function strains. Specifically, strains expressing piCas‐based plasmids show significantly higher l‐glutamate efflux compared to those carrying pBV2xp‐based plasmids. The piCas plasmid is based on the pNW33N backbone, which replicates via rolling‐circle replication, whereas pBV2xp uses a theta replication mechanism. These plasmids differ in copy number within *B. methanolicus* —approximately 15 copies for pNW33N and 6 copies for pBV2xp—which may contribute to the observed differences (Irla et al. 2016). A crucial distinction is the induction system controlling gene expression: pBV2xp relies on the gratuitous inducer xylose, which is not used as a carbon source by *B. methanolicus* , while piCas contains a mannitol‐inducible promoter regulating the expression of the *cas9* gene and the guide RNA cassette (Schultenkämper et al. 2019). The concentration of mannitol used to induce piCas was 2.5 g/L, which may itself stimulate increased l‐glutamate efflux in strains carrying this plasmid. Indeed, l‐glutamate efflux in the wild‐type strain growing in MVcM medium was approximately 3 mg/g CDW/h, increasing to around 60 mg/g CDW/h under MVcMii conditions (data not shown), which is consistent with the l‐glutamate efflux values found in the MGA3(pBV2xp) and MGA3(piCas) strains, respectively (Table 4). For RNA sequencing and to assess the media's impact on l‐glutamate production, the MVcMii medium, which contains 5 g/L mannitol, was a condition known to enhance l‐glutamate titers in *B. methanolicus* (Table 1). Therefore, the results presented in Table 4 compare each pair of strains individually—that is, the overexpression or repression plasmid against their respective empty vector counterparts—to account for these differences.

Overall, these results underscore the importance of media optimisation for enhancing l‐glutamate production in *B. methanolicus* . This organism has been shown to grow on extracts of the brown alga *Saccharina latissimi*, a promising next‐generation feedstock rich in mannitol (Hakvåg et al. 2020). The positive effect of mannitol supplementation alongside methanol broadens the range of viable feedstocks—namely seaweed and mannitol—supporting future industrial biotechnology efforts focused on circular bioeconomy and sustainable bioprocessing.

## 3 Conclusions

Here, we identify the small‐conductance mechanosensitive channel (MscS) of *B. methanolicus* as a pivotal l‐glutamate export system. This is evidenced by the regulation of fatty acid metabolism in *B. methanolicus* MGA3 under l‐glutamate overproduction conditions, which appears to trigger changes in membrane fluidity. Notably, the MscS in *B. methanolicus* exhibits a structural conformation similar to that in *E. coli* . Further evidence of its role comes from the suppression of l‐glutamate export with biotin‐replete conditions, and the modulation of l‐glutamate efflux through MscS overexpression and repression. These findings establish a foundation for engineering *B. methanolicus* for efficient l‐glutamate export and sustainable amino acid production.

## 4 Experimental Procedures

### 4.1 Strains and Growth Conditions

Unless stated otherwise, all chemicals used in this study were purchased from Sigma‐Aldrich. The *E. coli* strain DH5α (NEB 5‐alpha Competent *E. coli* —High Efficiency) served as the general cloning host, while the *B. methanolicus* wild‐type strain MGA3 (ATCC 53907) was employed both as the expression host and as a source of genetic material for molecular cloning. *E. coli* strains were routinely cultured at 37°C and 220 rpm in 125 mL Erlenmeyer flasks containing Lysogeny Broth (LB) or on LB plates (added with 15 g/L agar), both supplemented with 50 μg/mL kanamycin when harbouring pBV2xp‐ or piCas‐based plasmids. *B. methanolicus* grew at 50°C in plates containing 28 g/L SOB medium (supplied by Difco) added with 15 g/L agar. For growth experiments in this study, *B. methanolicus* strains were precultured at 50°C and 200 rpm in 500 mL flasks containing MVcMY, which is MvcM medium (Schendel et al. 1990) supplied with 250 mg/L yeast extract. Main cultures grew in Eppendorf New Brunswick Innova 42R incubators, either in 250 mL flasks or in Duetz system's flat plates of 24‐well and 11 mL volume (Kuhner Shaker). The defined growth media were MVcM or MVcMii (Table 5). The MVcMii medium was developed to enhance l‐glutamate production in *B. methanolicus* (Irla et al. 2025). As a standard practice, to ensure optimal cell fitness to obtain cell pellets for subsequent enzymatic assays and lipid content analysis, the cells were precultured and cultivated in SOB medium. All media were autoclaved at 120°C for 20 min; except for the MvcM vitamins and trace metals stocks (Table 5), which were sterile filtrated with 0.22 μm pore size filters. In all cultivations conducted in this study, bacterial cells were grown in a volume of media equivalent to 10% of their respective cultivating glassware volume. When needed, 10 g/L xylose was added for pBV2xp plasmid induction and 2.5 g/L mannitol was added for piCas plasmid induction. Kanamycin (25 μg/mL) was supplemented in media for *B. methanolicus* harbouring plasmids. Cultivations were performed in technical triplicates with a start optical density of 0.2. Growth was monitored by measuring optical density with a cell density meter (WPA CO 8000 Biowave). The optical density values were converted to g CDW according to Myers et al. (2013).

**TABLE 5:** MVcM and MVcMii media composition.

	MVcM	MVcMii
***Media buffer***		
**Component**	**Concentration**	
K_2_HPO_4_	4.1 g/L	4.1 g/L
NaH_2_PO_4_	1.3 g/L	1.3 g/L
(NH_4_)_2_SO_4_	2.1 g/L	4.0 g/L
MOPS	—	20.9 g/L
*Vitamins stock (1000×)	1.0 mL/L	1.0 mL/L
MgSO_4_ 1 M	1.0 mL/L	0.2 mL/L
^#^Trace metals stock (1000×)	1.0 mL/L	1.0 mL/L
Methanol absolute	8.1 mL/L	8.1 mL/L
Mannitol	—	5.0 g/L
* ***Vitamins stock (1000*** ×)		
**Component**	**g/L**	
d‐Biotin	0.100	
Thiamine*HCl (vitamin B1)	0.100	
Riboflavin (vitamin B2)	0.100	
Pyridoxine*HCl (vitamin B6)	0.100	
Pantothenate (vitamin B5)	0.100	
Nicotinamide (vitamin B3)	0.100	
p‐Aminobenzoic acid (vitamin L1)	0.020	
Folic acid (vitamin B11)	0.010	
Cyanocobalamin (vitamin B12)	0.010	
Lipoic acid (thioctic acid)	0.010	
^**#**^ ***Trace metals stock (1000*** ×)		
**Component**	**g/L**	
FeSO_4_*7H_2_O	5.56	
CuCl_2_*2H_2_O	0.027	
CaCl_2_*2H_2_O	7.35	
CoCl_2_*6H_2_O	0.040	
MnCl_2_*4H_2_O	9.90	
ZnSO_4_*7H_2_O	0.288	
Na_2_MoO_4_*2H_2_O	0.048	

*Note:* The media buffer pH was adjusted to 7.0 with NaOH before autoclaving. Sterile‐filtered concentrated vitamin and trace metal solutions were added at a 1:1000 dilution after autoclaving.

To specifically target the *B. methanolicus* cell membrane, we first pre‐cultured the MGA3 strain overnight in shake flasks containing MVcMY medium. The cultures were then transferred to shake flasks containing MVcM supplemented solely with cyanocobalamin (vitamin B12) as a vitamin source. The optical density was adjusted to 0.1. Once the cultures reached an optical density of approximately 2.0, they were used to inoculate triplicates in Duetz system plates. The media contained cyanocobalamin and varying concentrations of biotin (0.1, 2, 4, 6, 8, or 10 mg/L) as a replacement for the vitamin mix (Table 5).

### 4.2 Molecular Cloning

For overexpression of the *mscS* gene, we selected the theta‐replicating plasmid pBV2xp, which was optimised for controlled gene expression of *B. methanolicus* MGA3 by means of a *Bacillus megaterium* ‐derived xylose catabolism *xylR* regulator and a xylose‐inducible *xylA* promoter in its sequence (Irla et al. 2016; Drejer et al. 2020). The plasmid underwent restriction with the *Bam*HI enzyme (New England Biolabs–NEB), and the *mcsC* gene was amplified from *B. methanolicus* MGA3 genomic DNA using forward primer 5′‐aagggggaaatggctATGAAAGCTGGTGATTTTATGCAAG‐3′ and reverse primer 5′‐ctcatggtacggatcCTATTCCTTTCCTAACTGTTTTTGC‐3′, both containing overlapping regions (lowercase) with the *Bam*HI cutting site of the pBV2xp plasmid. Furthermore, in order to obtain *mscS* gene repression in *B. methanolicus* MGA3, its genome sequence (GenBank: CP007739) was used as a basis for 20 nt gene target single‐guided RNA (sgRNA) sequence selection (antisense strand) by the CRISPy‐web tool (Blin et al. 2016). Next, the piCas plasmid, established by Schultenkämper et al. for gene repression in *B. methanolicus* MGA3 (Schultenkämper et al. 2019), served as the DNA template for PCR, employing forward primer 5′‐aaatgaagatacatggatcgcgatGTTTTAGAGCTAGAAATAGCAAGTTA‐3′ and reverse primer 5′‐atcgcgatccatgtatcttcatttATATTTATCATAAAACGTTTATATCCC‐3′. This enabled the amplification of the plasmid with overlapping regions comprising the *mscS* 20 nt sgRNA sequence (represented in lowercase in the primer sequences). Polymerase chain reaction (PCR) products were amplified using CloneAmp HiFi PCR Premix (Takara) and purified using a QIAquick PCR Purification Kit from Qiagen. *B. methanolicus* MGA3 genomic DNA was isolated using the NEB's Monarch Genomic DNA Purification kit. Plasmid DNA was isolated using the Plasmid Miniprep–Classic kit (Zymo Research). Plasmids were constructed by overlapped end joining with the Gibson assembly method (Gibson et al. 2009), employing the NEBuilder HiFi DNA Assembly kit. Constructed plasmids were introduced into chemically competent *E. coli* DH5α cells (Hanahan 1983). The procedures above were done following the manufacturer's recommendations. The sequences of cloned DNA fragments were confirmed by Sanger sequencing elsewhere (Eurofins Genomics). The plasmid constructed for *mscS* gene expression was named pBV2xp‐*mscS*, and the plasmid constructed for *mscS* repression was named piCas‐*mscS*. *B. methanolicus* MGA3 was made electrocompetent and transformed by electroporation as described previously (Jakobsen et al. 2006). The novel *B. methanolicus* strains were designated as MGA3(pBV2xp‐*mscS*) and MGA3(piCas‐*mscS*). They were consistently compared to their previously constructed empty vector control counterparts, namely, MGA3(pBVxp) and MGA3(piCas) (Schultenkämper et al. 2019; Drejer et al. 2020).

### 4.3 RNA Sequencing and Bioinformatics

We conducted differential gene expression analysis through RNA sequencing, comparing the transcriptomes of *B. methanolicus* MGA3 growing in either MVcM or MVcMii (Table 5). Each condition involved six replications of *B. methanolicus* cultures, cultivated in shake flasks with minimal media until reaching mid‐exponential phase (optical density of approximately 0.8). Subsequently, three replications for each cultivation condition were harvested by centrifugation at 7197 × *g* and 4°C for 10 min, followed by fast‐freezing in liquid N_2_ and stored at −80°C for later total RNA isolation. The remaining triplicates were further monitored to assess growth and l‐glutamate production parameters.

Total RNA extraction from *B. methanolicus* MGA3 cells was performed using the NucleoSpin RNA isolation kit (Machery‐Nagel, Düren, Germany) and the RNase‐free DNase set (Qiagen, Hilden, Germany), following the manufacturer's instructions. RNA purity was assessed with a NanoDrop Spectrophotometer (Thermo Scientific). Furthermore, to assess the presence of contaminating DNA, the RNA material was tested using gene‐specific primers 5′‐GTGACCACAAATAAGAAAAAACTTACTACAAGC‐3′ and 5′‐TTAAACTTTCTTTTGTACAGGTAAACCTAGAC‐3′ for the amplification of the gene *katA* using the GoTaq DNA polymerase (Promega). No amplification product was observed for any of the tested samples (data not shown). Isolated RNA samples from *B. methanolicus* MGA3 were sent for RNA sequencing elsewhere (Eurofins genomics). The RNA material was analysed using the 2100 Bioanalyzer (Agilent Technologies), and rRNA depletion was carried out for each individual sample with the NEBNext rRNA Depletion Kit for bacteria (New England BioLabs). Equal amounts of triplicates from each condition were pooled together, and total RNA was then utilised for strand‐specific cDNA library preparation employing Illumina's Genome Sequencer technology in NovaSeq 6000 S4 PE150 XP mode.

Prior to mapping of the generated reads onto the reference genome, the RNA sequencing raw sequences were trimmed using the tool Trimmomatic version 0.4 to a minimal length of 35 base pairs (Bolger et al. 2014). The trimmed reads were mapped to the *B. methanolicus* MGA3 reference sequences of the chromosome as well as the two plasmids pBM19 and pBM69 (GenBank accession numbers CP007739, CP007741, and CP007740, respectively) using the software for short read alignment Bowtie2 (Langmead and Salzberg 2012). For the visualisation of the mapped reads the ReadXplorer software was used (Hilker et al. 2014). The differential gene expression analysis was performed with the statistical method DESeq (Anders and Huber 2010) using the same software. In order to designate a gene as differentially expressed, the cut‐off values were set to a change in expression level higher than 30, for which the *p*‐value was adjusted to be equal to or less than 0.01. Sequences of differentially expressed genes that coded for proteins of unknown function were subjected to BLASTx analysis for identification of protein family conservations (Altschul et al. 1990). The STRING database v12.0 was used to perform gene ontology and protein–protein interaction analyses of the significant differentially expressed genes (Szklarczyk et al. 2019). The RNA‐seq data generated in this study have been deposited in the NCBI Gene Expression Omnibus (GEO) under accession number GSE301205. Aligned reads reads (BAM files) with associated metadata are available at https://www.ncbi.nlm.nih.gov/geo/query/acc.cgi?acc=GSE301205.

A phylogenetic analysis was performed using the MPI Bioinformatics Toolkit (Zimmermann et al. 2018) as follows. First, the amino acid sequence of MGA3 MscS (UniProt I3EBZ0) was searched against the SwissProt database using PSI‐BLAST (Altschul et al. 1997) with the BLOSUM45 scoring matrix. The resulting hits were clustered using MMseqs2 (Steinegger and Söding 2017), grouping sequences with 80% sequence identity into the same cluster. Representative sequences from each cluster were aligned using Clustal Omega (Sievers et al. 2011), and a phylogenetic tree was generated using the maximum‐likelihood method with the LG model of amino acid replacement and 100 bootstrap replicates, implemented in PhyML 3.0 (Guindon et al. 2010). The resulting tree was visualised using FigTree (https://github.com/rambaut/figtree).

### 4.4 Analysis of Culture Supernatants by Means of High‐Pressure Liquid Chromatography

For the analysis of amino acid concentrations, 1 mL of the culture supernatant was obtained from the bacterial cultures by centrifugation for 15 min at 7197 × *g*. Supernatants were routinely collected after 24 h of growth, except for the samples used to measure l‐glutamate efflux, which were taken two hours after plasmid induction. Extracellular l‐glutamate was quantified by means of high‐pressure liquid chromatography (HPLC, Waters Alliance e2695 Separations Module). The samples underwent FMOC‐Cl (fluorenylmethyloxycarbonyl chloride) derivatisation before the analysis, according to the protocol described before (Melucci et al. 1999), and were separated on a column (Symmetry C18 Column, 100 Å, 3.5 μm, 4.6 mm × 75 mm, Waters) according to the gradient flow established by Brito et al., with elution buffers comprising 50 mM Na‐acetate pH = 4.2 and an organic solvent, acetonitrile (Brito et al. 2021). The detection was performed with a Waters 2475 HPLC Multi Fluorescence Detector (Waters), with excitation at 265 nm and emission at 315 nm.

### 4.5 α‐Ketoglutarate Dehydrogenase Assay

We cultivated the *B. methanolicus* strains MGA3(pBVxp), MGA3(pBV2xp‐*mscS*), MGA3(piCas), and MGA3(piCas‐*mscS*) in shake flasks containing SOB medium. The cells were grown to mid‐exponential phase (optical density between 0.8 and 1.5). Ten millilitres of each culture were transferred to Falcon tubes and centrifuged at 7197 × *g* and 4°C. The cell pellets were washed twice under the same centrifugation conditions using 1 mL ice‐cold KGDH Assay Buffer from the α‐Ketoglutarate Dehydrogenase Activity Colorimetric Assay Kit (Sigma‐MAK189). Washed cells (resuspended in 1 mL) were transferred to screw cap microcentrifuge tubes, and 250 μL of glass beads (0.75–1 mm, Roth) were added. Crude extracts were obtained using a Retsch MM 400 mixer mill for 30 s at 6.5 m/s. The tubes were placed on ice for 5 min before repeating the process. Protein concentration in crude extracts was determined by means of Bradford assay (Bradford 1976), using bovine albumin serum as standard. The α‐KGDH activity was measured colorimetrically using the kit manufacturer's guidelines, in which the generation of NADH in the samples is measured based on provided standards. For the assay, a 96‐well flat‐bottom plate was used, and the absorbance at 450 nm of the samples was measured in a TECAN‐Infinite M200‐Microplate reader. One unit of α‐KGDH activity represented 1 nmol of NADH generated per minute of reaction per mg protein.

### 4.6 Lipid Analysis

To extract lipids from *B. methanolicus* cells, the strains MGA3(pBV2xp), MGA3(pBV2xp‐*mscS*), MGA3(piCas), and MGA3(piCas‐*mscS*) were cultivated in shake flasks containing SOB medium until reaching an optical density of 0.8–1.5. The cells were centrifuged at 7197 × *g* for 15 min, and cell pellets were stored at −80°C until further use. The cells were thawed on ice and washed twice in PBS buffer (pH 7.4; Thermo Scientific), with centrifugation at 1878 × *g* for 5 min each time. The washed pellets were resuspended in 500 μL of a methanol mixture (2:1 (v/v)) to proceed with lipid extraction following the Bligh and Dyer method (Bligh and Dyer 1959).

Lipid profiling was carried out as described elsewhere (Bartosova et al. 2021), using a UHPSFC (ultra‐high performance supercritical fluid chromatography) system coupled with a SYNAPT G2‐S HDMS hybrid quadrupole orthogonal time‐of‐flight mass spectrometer (Waters, Milford, MA, USA). Separation utilised an Acquity BEH UPC2 column (100 mm × 3 mm, 1.7 μm) paired with a VanGuard precolumn (BEH 2.1 × 5 mm, Waters). The column temperature was maintained at 50°C, with a flow rate of 1.9 mL/min, and the automated back‐pressure regulator (ABPR) set at 1800 psi. A methanol solution (99:1, v/v) containing 30 mM ammonium acetate served as the modifier. The gradient profile was programmed as follows: 0 min, 1%; 4 min, 30%; 4.4 min, 50%; 6.25 min, 50%; 7.25 min, 50%; 7.35 min, 1%; and 8.50 min, 1%. A methanol:isopropanol mixture (50:49:1 (v/v/v)) was used as a make‐up liquid, with a flow rate of 0.2 mL/min. The mass spectrometer operated in MSE mode with collision energy ramping from 20 to 30 eV. Data were collected within a mass range of 50–1200 Da at a resolution of 20,000. Positive ion electrospray ionisation was used, with settings as follows: capillary voltage at 3.0 kV, source temperature at 150°C, sampling cone voltage at 40 V, source offset at 60 V, desolvation temperature at 500°C, cone gas flow at 50 L/h, desolvation gas flow at 900 L/h, and nebulizer gas pressure at 4 bar. Leucine enkephalin was employed as the lock mass. Principal Component Analysis was performed to explore patterns of variation in lipid abundances across the different strains. The lipid abundance data, obtained in triplicates for each strain, were scaled to ensure that all variables had equal influence on the analysis.

### 4.7 Modelling and Analysis of MscS 3D Structure

The 3D coordinates of MGA3 MscS quaternary structure were predicted using the AlphaFold 3 web server (Abramson et al. 2024). The input was seven copies of the primary sequence of MGA3 MscS (UniProt I3EBZ0). The *E. coli* mechanosensitive transporter MscS in the closed (PDB 6PWN) and open (PDB 2VV5) conformations was used as a reference to guide the computational analysis of the studied protein. Considering the open structure has shorter termini and to focus the analysis on the relevant part of the proteins, for both the closed reference and the target protein, the two termini were shortened for each monomer if necessary; in the case of MGA3 MscS, the truncated regions were 1–20 in the *N*‐terminus and 287–300 in the C‐terminus, whereas for *E. coli* MscS, only the *N*‐terminus needed to be shortened by truncating the residues 1–24.

Since the predicted structure for the MGA3 transporter is in the closed conformation, the *E. coli* MscS closed structure was used as a reference. The proteins were inserted into a model bilayer by using the webserver OPM (Lomize et al. 2022), where specific membrane models were chosen for each protein, i.e., the inner membrane of Gram‐negative bacteria for the *E. coli* transporter and the membrane of Gram‐positive bacteria for the *B. methanolicus* transporter. The output result was used as input for the explicit membrane anisotropic normal mode analysis (exANM) performed using the Python library Prody (Lezon and Bahar 2012).

For the *E. coli* transporter, the radius of the membrane nodes was kept at the default value of 3.1, while for the target MGA3 transporter both the values 3.1 and 7.0 were used to change the properties of the membrane. The value of 7.0 effectively reduces the density of lipid nodes in the model. The thickness of the lipidic layer was set by the values membrane_low at −20 and membrane_high at 20 in all cases to guarantee the inclusion of the transmembrane part in the bilayer. The hull was set to False to avoid curvature of the membrane, and the elementary unit of the elastic network was set to the default geometry, which is face‐centered‐cubic (FCC). With these parameters, 100 normal modes were computed based on the Cα of the proteins, but only the first three were taken for further analysis.

To identify the most relevant normal modes, the deformation vector of the opening mechanism was computed for *E. coli* transporter by using the crystal structure of the closed and open conformations. The function calcDeformVector defined in Prody was used to compute the deformation vector by using only one monomer from the closed and open conformations since the motion is symmetrical along the seven monomers. To compare the displacement of the deformation vector and the normal modes, the normalised square fluctuations were computed by using the function calcSqFlucts implemented in Prody. The input of this function can be a normal mode which is already normalised or the deformation vector after normalisation. Detailed information on the computational analysis can be found in https://github.com/gcourtade/papers/tree/master/2025/MscS‐transporter.

## Author Contributions

**Luciana Fernandes Brito:** conceptualization, investigation, formal analysis, visualization, writing – original draft, writing – review and editing, validation, funding acquisition. **Davide Luciano:** investigation, formal analysis, visualization, writing – original draft, writing – review and editing. **Marta Irla:** conceptualization, writing – review and editing, funding acquisition. **David Virant:** investigation, funding acquisition. **Gaston Courtade:** writing – review and editing, supervision, funding acquisition. **Trygve Brautaset:** conceptualization, writing – review and editing, project administration, funding acquisition.

## Conflicts of Interest

The authors declare no conflicts of interest.

## Supporting information


**Data S1:** mbt270252‐sup‐0001‐FigureS1‐S5‐TableS1.pdf.

## Data Availability

The data that support the findings of this study are openly available in Gene Expression Omnibus at https://www.ncbi.nlm.nih.gov/geo/query/acc.cgi?acc=GSE301205, reference number GSE301205.
