# Supplementary material for: Identification of MscS as a Key L‐Glutamate Exporter in Bacillus methanolicus
Source: Microb Biotechnol. 2025 Oct 22;18(10):e70252. doi: 10.1111/1751-7915.70252 (PMC12541555; doi:10.1111/1751-7915.70252)
Supplement: Supplementary file 1 — Data S1: mbt270252‐sup‐0001‐FigureS1‐S5‐TableS1.pdf. [file MBT2-18-e70252-s001.pdf]

SUPPLEMENTARY MATERIAL

# Identification of MscS as a Key L-Glutamate Exporter in *Bacillus methanolicus*

Luciana Fernandes de Brito[1*], Davide Luciano[1], Marta Irla[2], David Virant[3], Gaston Courtade[1], Trygve Brautaset[1]

[1]Department of Biotechnology and Food Science, Norwegian University of Science and Technology, 7491 Trondheim, Norway

[2]Department of Biological and Chemical Engineering, Aarhus University, 8000 Aarhus, Denmark

[3]Acies Bio d.o.o., Tehnološki Park 21, SI-1000 Ljubljana, Slovenia

*Corresponding author: luciana.f.d.brito@ntnu.no

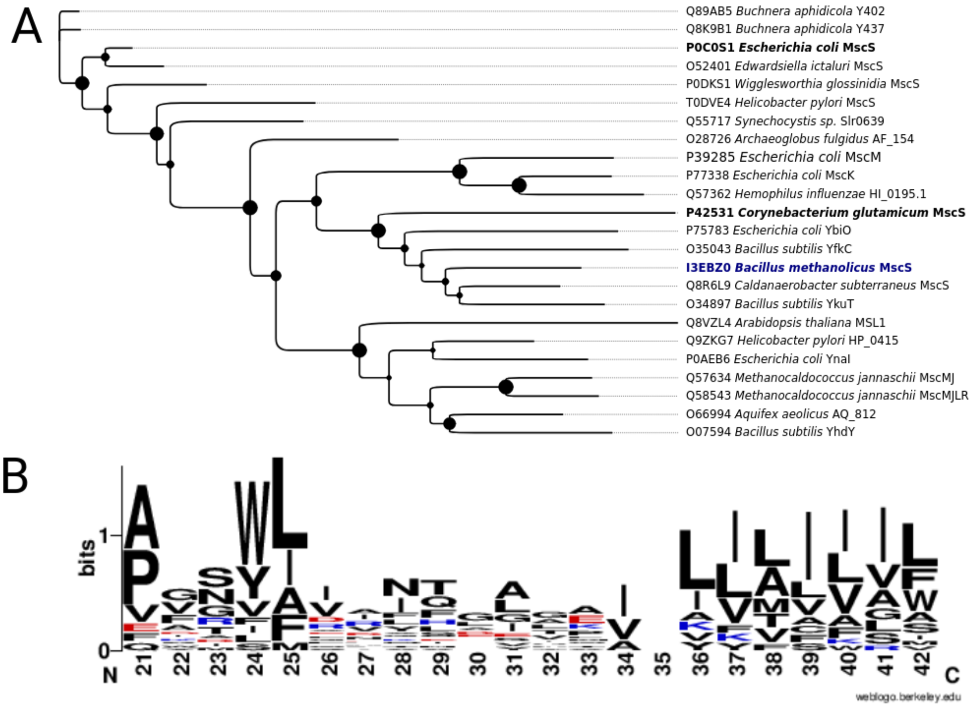


**Figure S1.** A) Phylogenetic tree built using the maximum likelihood method using *Bacillus methanolicus* MGA3 MscS (blue). *Escherichia coli* MscS and *Corynebacterium glutamicum* MscS are emphasized to facilitate comparison with these proteins. The UniProt accession codes for all proteins are included. The size of the black circles at the branching points is proportional to the bootstrap values, ranging from 20 to 100, where 100 is the maximum. The bootstrap values are indicative of the node quality. B) Sequence logo generated from the multiple sequence alignment of the first transmembrane helix of each protein in Panel A. Positively charged amino acids (R and K) are coloured blue, negatively charged amino acids (E and D) are coloured red, and all other amino acids are coluored black.

**Table S1.** Geometric and thermodynamic parameters of the membrane complex predicted by OPM server.

| Transporter | Depth/Hydrophobic Thickness | ΔGtransfer | TILT ANGLE |
|---|---|---|---|
| *E. coli* MscS | 31.3 ± 0.8 Å | -133.9 Kcal/mol | 0.0 ± 0° |
| *B. methanolicus* MscS | 33.1 ± 0.7 Å | -157.4 Kcal/mol | 0.0 ± 0° |

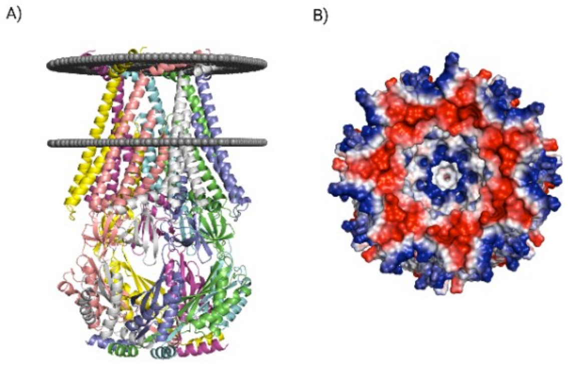


**Figure S2.** A) Full-length *B. methanolicus* MGA3 MscS transporter structure predicted with AlphaFold 3. B) Electrostatic map of the top view of the transporter which shows a positively rich channel. The vacuum electrostatic map was computed using PyMOL (https://www.pymol.org/).

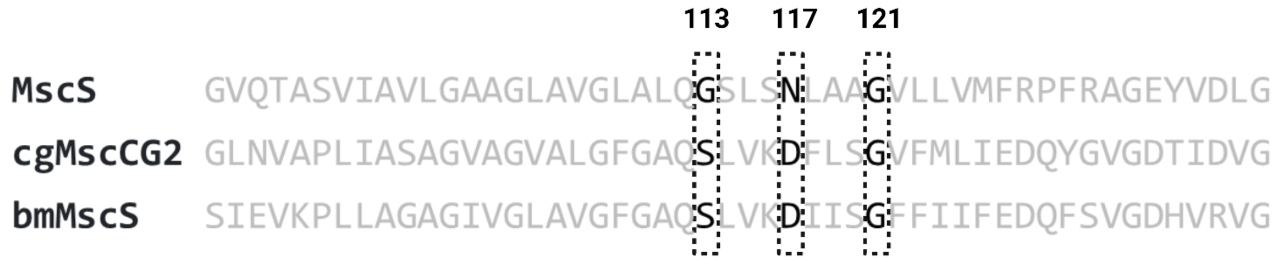


**Figure S3.** Multiple sequence alignment of the TM3 region of the *E. coli* MscS transporter (MscS, NCBI: BAE76988), the *C. glutamicum* MscCG2 transporter (cgMscCG2, NCBI: WP_011014245), and the *B. methanolicus* MscS-like transporter (bmMscS, NCBI: WP_003347065).

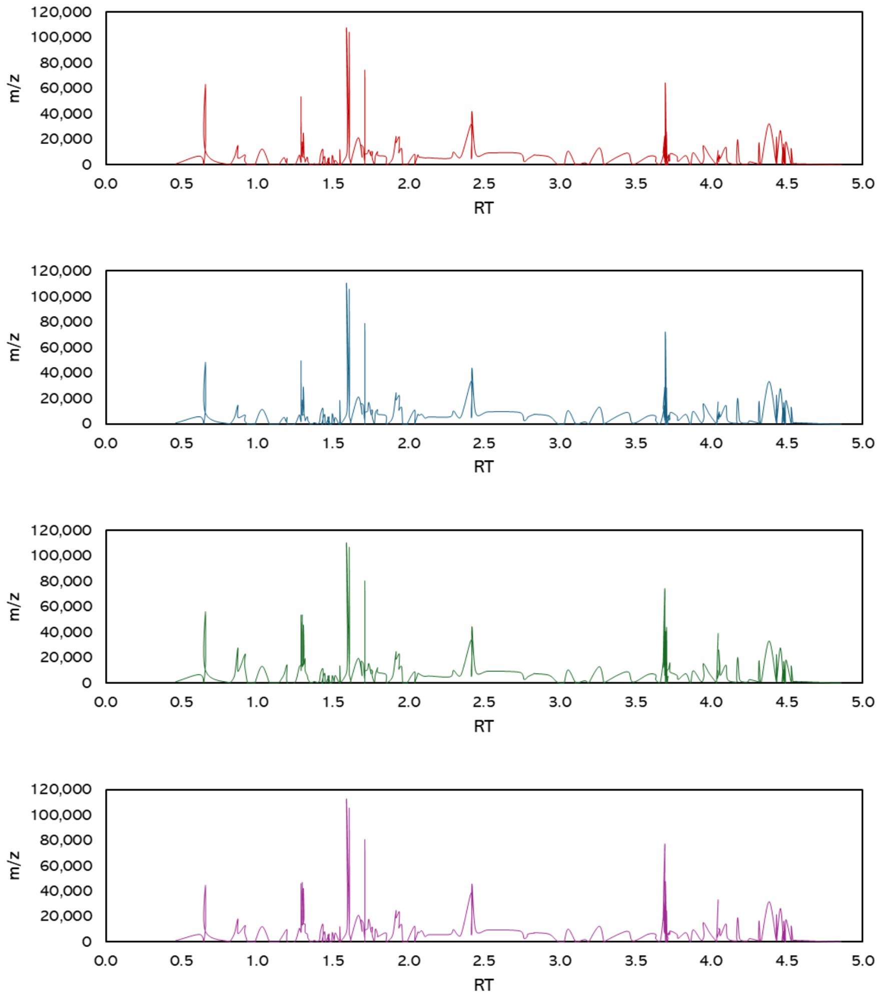


**Figure S4.** Lipid profiling chromatogram of *B. methanolicus* strains MGA3(pBV2xp), MGA3(pBV2xp-*mscS*), MGA3(piCas) and MGA3(piCas-*mscS*) (ordered from top to bottom).

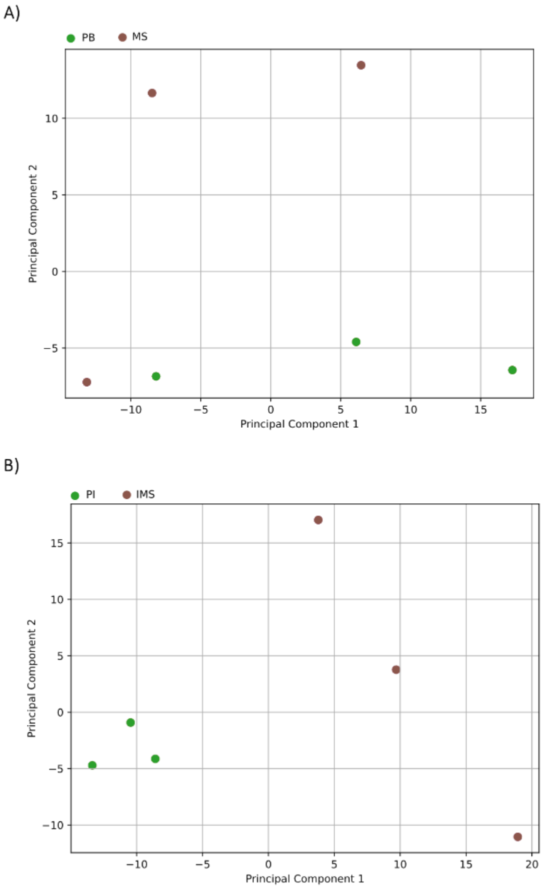


**Figure S5.** Principal component analysis of the lipid mass abundances detected by means of UHPSFC analysis of the lipid extracts from *B. methanolicus* strains PB- MGA3(pBVxp), in comparison to MS- MGA3(pBV2xp-*mscS*) (A), and PI- MGA3(piCas), in comparison to IMS-MGA3(piCas-*mscS*) (B). Component 1 refers to lipid abundances across triplicates of the tested strains and component 2 refers to lipid masses (m/z) detected during the UHPSFC analysis.
